# Refined target-mediated drug disposition modeling of the anti-tissue factor pathway inhibitor antibody MG1113 in cynomolgus monkeys and rabbits

**DOI:** 10.3389/fphar.2025.1745702

**Published:** 2026-01-07

**Authors:** Heechun Kwak, Yoo-Seong Jeong, Jiyoung Kim, Minsoo Lee, Seonyoung Byoun, Yasunori Aoki, Suk-Jae Chung, Wooin Lee

**Affiliations:** 1 College of Pharmacy and Research Institute of Pharmaceutical Sciences, Seoul National University, Seoul, Republic of Korea; 2 Research and Early Development Department, GC Biopharma Corp., Yongin-si, Republic of Korea; 3 Laboratory of Quantitative System Pharmacokinetics/Pharmacodynamics, Josai International University, Tokyo, Japan; 4 Drug Metabolism and Pharmacokinetics, Research and Early Development, Cardiovascular, Renal and Metabolism (CVRM), BioPharmaceuticals R&D, AstraZeneca, Gothenburg, Sweden

**Keywords:** antibody, cluster Gauss-Newton method, MG1113, pharmacokinetic modeling, target-mediated drug disposition

## Abstract

**Introduction:**

MG1113 is a humanized immunoglobulin G4 antibody targeting the Kunitz-type protease inhibitor 2 domain of tissue factor pathway inhibitor (TFPI) and is under clinical investigation for hemophilia treatment. This study aimed to refine a previously developed target-mediated drug disposition (TMDD) model for MG1113 by incorporating both targets [e.g., soluble TFPI-α (sTFPI-α) and membrane-bound TFPI (mTFPI)] and a transit compartment to capture delayed absorption after subcutaneous (s.c.) dosing.

**Methods:**

The refined TMDD model was fitted to the plasma profiles of MG1113 and sTFPI-α in cynomolgus monkeys that received various intravenous and s.c. doses of MG1113 using the Cluster Gauss-Newton Method (CGNM). The optimized model parameters were scaled allometrically and used to simulate the concentration-time profiles of MG1113 and sTFPI-α in rabbits and humans.

**Results:**

The refined TMDD model provided an improved model performance overall, compared to the previous model when fitted to monkey data. When extrapolated to rabbits, the model prediction showed a good agreement with the observed MG1113 and sTFPI-α data, supporting its interspecies applicability. In humans, the model prediction suggested that maintaining sTFPI-α suppression below 25% of baseline, a level associated with therapeutic efficacy, could be achieved with a weekly dose of 3.3 mg/kg MG1113.

**Conclusion:**

The refined TMDD model better characterized the nonlinear pharmacokinetic and pharmacodynamic profiles of MG1113 across species by incorporating both targets and delayed absorption after s.c. dosing. This model enabled quantitative prediction of sTFPI-α suppression in relation to MG1113 dose and baseline target levels, supporting a rational dose selection for ongoing and future clinical studies.

## 1 Introduction

Tissue factor pathway inhibitor (TFPI) serves as a key negative regulator in the coagulation pathway. TFPI binds to activated factor X (FXa), and the resulting TFPI/FXa complex suppresses the extrinsic FXase complex composed of tissue factor and activated factor VII (Supplementary Figure S1). The biology of TFPI is complex in that it exists in structurally distinct isoforms, namely, TFPI-α, TFPI-β, and truncated TFPI-α, which are distributed across three physiological pools. The largest pool (∼85% of total TFPI) is associated with endothelial cells, including TFPI-α linked to cell surface glycosaminoglycan, TFPI-β anchored on the cell surface via a glycosylphosphatidylinositol (GPI) co-receptor, and intracellular TFPI-α (Hansen et al., 2014; Yuan et al., 2019). A second pool (<10%) circulates in plasma and consists primarily of TFPI-α in a truncated, lipoprotein-bound form. The remaining ∼5% resides in platelets as TFPI-α (Broze and Girard, 2012). These isoforms and pools represent two major states of TFPI: a membrane-bound form (mTFPI) and a soluble form circulating in plasma (sTFPI). Soluble form TFPI-α (sTFPI-α) in plasma is often used as a pharmacodynamic (PD) marker to guide dosing strategies, typically aiming to reduce sTFPI-α levels to below 25% of baseline for the desired pharmacological effect (Chowdary et al., 2015; Eichler et al., 2018).

In recent years, TFPI has emerged as a novel therapeutic target in the management of bleeding disorders. In 2024, the US FDA approved two TFPI-targeting monoclonal antibodies, concizumab and marstacimab, for the treatment of hemophilia (Jiang et al., 2025). Another TFPI-targeting antibody in clinical development is MG1113, a humanized immunoglobulin G4 (IgG4) antibody that binds to the Kunitz-type protease inhibitor 2 (K2) domain of TFPI. By binding to both sTFPI-α and mTFPI, MG1113 has been shown to promote blood coagulation in rabbit models of hemophilia and in plasma obtained from patients with hemophilia (Kwak et al., 2020). MG1113 is currently being investigated in early-phase clinical trials (NCT03855696 and NCT05493631).

The pharmacokinetic (PK) profiles of therapeutic antibodies are often influenced by saturable interactions with their pharmacological targets, a phenomenon known as target-mediated drug disposition (TMDD) (An, 2020). A previous PK model developed for MG1113 incorporated TMDD by considering sTFPI-α (measured in plasma) as the sole target (Kwak et al., 2021). However, given that sTFPI-α represents less than 10% of total TFPI, it is important to consider interactions between MG1113 and mTFPI, which constitutes the predominant pool. Indeed, TFPI-β, a major component of mTFPI, contains the K2 domain targeted by MG1113, and the abundance of TFPI-β (∼170 nmol in humans) is substantially higher than that of sTFPI-α (∼4.8 nmol in humans) (Girard et al., 2012). As a precedent, the PK profile of concizumab was successfully described by a model that incorporated interactions with both mTFPI and sTFPI, with mTFPI-mediated endocytosis playing a major role in its nonlinear PK behaviors (Yuan et al., 2019). Another aspect of refining the previous model for MG1113 is its feedback component, which accounts for the rebound observed in plasma sTFPI-α levels (more evident in the profiles of individual monkeys, Supplementary Figure S2). A rebound phenomenon is known to occur when the internalization rate of the drug-target complex is slower than the elimination rate of the free drug and the degradation rate of the free target (Aston et al., 2014; Aston et al., 2017). These rate processes can be described by typical TMDD principles, and mechanistic modeling of sTFPI-α rebound through rate constants that correspond to specific biological processes may enhance biological interpretability and interspecies predictability, which are often difficult to achieve when utilizing a non-mechanistic feedback compartment.

The current study reports the findings from a refined TMDD model of MG1113, which incorporates its saturable interactions with both targets (e.g., sTFPI-α and mTFPI) and a transit compartment for its subcutaneous (s.c.) absorption. Kinetic modeling was performed using the Cluster Gauss-Newton Method (CGNM), an algorithm that identifies multiple sets of approximate solutions to nonlinear least-squares problems (Aoki et al., 2022). The refined model was fitted to MG1113 and sTFPI-α data observed in monkeys to estimate model parameters, which were subsequently extrapolated to rabbits and humans via allometric scaling. Simulated concentration-time profiles in rabbits were in good agreement with the observed data. Finally, the model was applied to simulate plasma profiles of MG1113 and sTFPI-α in humans under clinically relevant dosing regimens.

## 2 Materials and methods

### 2.1 Data sources

The current study reanalyzed previously published data on plasma MG1113 and sTFPI-α concentrations in cynomolgus monkeys (Kwak et al., 2021). The monkeys received MG1113 at one of three dose levels via either intravenous (i.v.) or s.c. administration: 2.5, 5.0, and 10.0 mg/kg (17.2, 34.4, and 68.8 nmol/kg). As the body weight data for individual monkeys were not available, we used a representative body weight of 3.5 kg as reported in the literature (Zhao et al., 2015) to calculate the administered amount. For modeling, the dose was converted to the amount administered to a monkey weighing 3.5 kg (8.75, 17.5, or 35 mg, equivalent to 60.2, 120.4, or 240.8 nmol). Measured plasma concentrations of MG1113 and sTFPI-α were converted to molar units using their respective molecular weights of 145.36 and 35 kDa (Kwak et al., 2021). The raw data, provided by GC Biopharma, were generated using enzyme-linked immunosorbent assays (ELISA) that detected free forms of MG1113 and sTFPI-α.

### 2.2 Structure and model parameters of the refined TMDD model for MG1113

**FIGURE 1:**
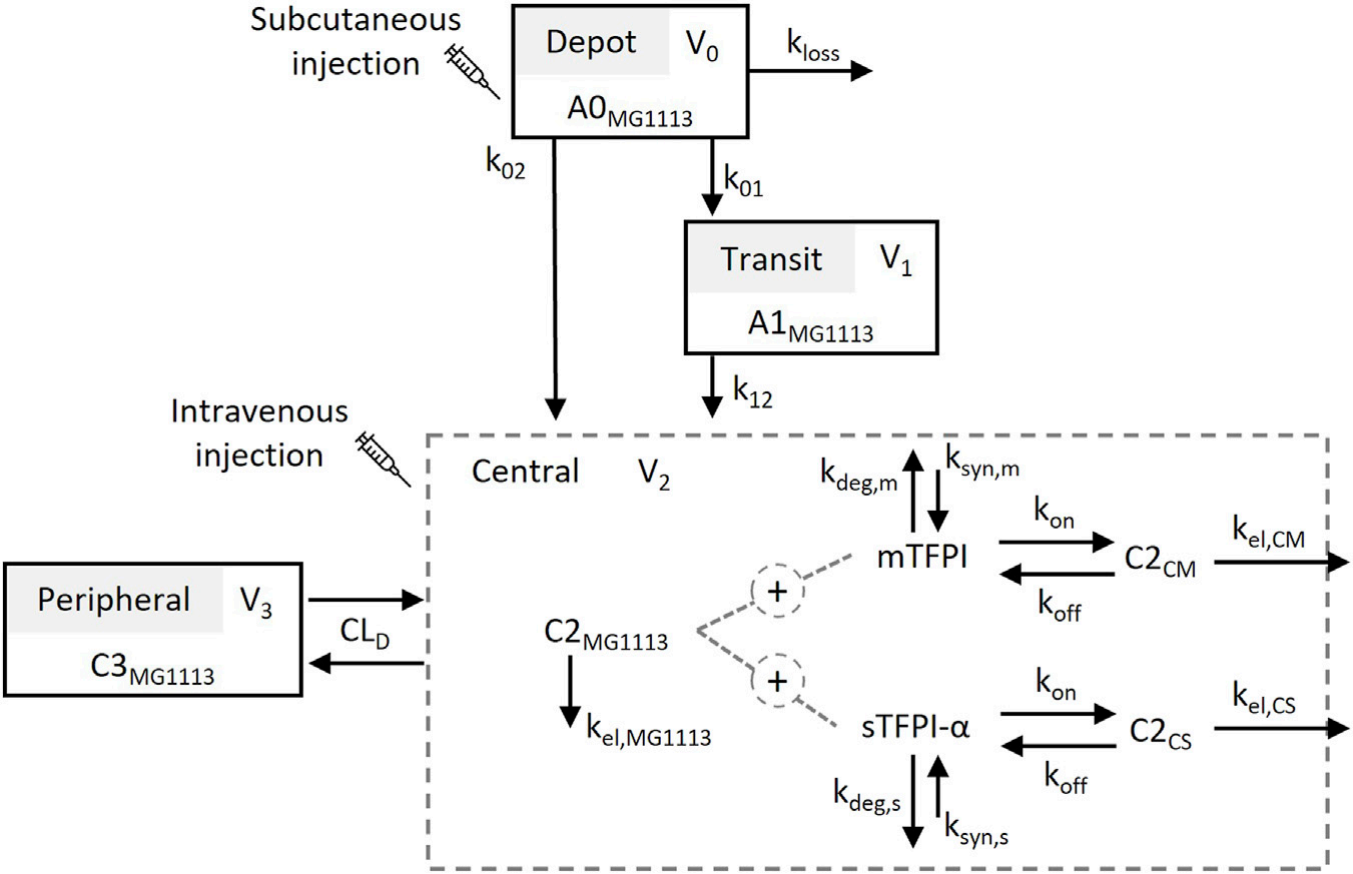
Structure of the refined TMDD model for MG1113 in the current study. Refer to the text and Table 1 for abbreviations and detailed descriptions.

Our strategies for refining the previously published TMDD model of MG1113 focused on two aspects: (i) revision of the target binding and feedback components for enhanced biological interpretability and interspecies scaling, and (ii) addition of a transit compartment to better capture the delayed absorption after s.c. administration. In this study, we assumed that TFPI circulating in plasma consists predominantly of the alpha isoform (sTFPI-α). In addition, we considered the primary membrane-bound forms of TFPI (mTFPI) capable of interacting with circulating MG1113 to be GPI-anchored TFPI-β (mTFPI-β) and TFPI-α bound to cell surface glycosaminoglycan (mTFPI-α), both of which harbor the K2 domain, the binding site for MG1113. The refined model structure is presented in Figure 1, and the model equations are described in the Supplementary Materials. The detailed parameter descriptions are summarized in Table 1.

**TABLE 1:** List of the fixed and optimized parameters and summary of the accepted parameter values by the CGNM analysis.

Parameter (unit)	Description	Values	Accepted parameter	
			Rank 1	Median (min, max)
Fixed parameter		Fixed values		
BW (kg)	The monkey’s body weight	3.5 (Zhao et al., 2015)	-	-
K_D_ (nM)	The equilibrium dissociation constant of MG1113/TFPI complex	0.04665 (Kwak et al., 2020)	-	-
Optimized parameter		Initial Range (min, max)		
CL_D_ (L/day)	Intercompartment clearance of MG1113	(0.002755, 27.55) (Kwak et al., 2021)	0.1454	0.1456 (0.1453, 0.1458)
V_2_ (L)	Central compartment volume	(0.00157, 15.7) (Zhao et al., 2015)	0.1112	0.1112 (0.1111, 0.1113)
V_3_ (L)	Peripheral compartment volume	(0.001047, 10.47) (Kwak et al., 2021)	0.1346	0.1346 (0.1346, 0.1348)
k_01_ (1/day)	Depot to transit compartment distribution rate constant	(0.01, 100)	20.32	20.30 (20.26, 20.37)
k_02_ (1/day)	Depot to central compartment distribution rate constant	(0.01, 100)	8.859 × 10^−8^	1.549 × 10^−7^ (3.052 × 10^−10^, 9.553 × 10^−6^)
k_12_ (1/day)	Transit to central compartment distribution rate constant	(0.01, 100)	0.4033	0.4034 (0.4033, 0.4037)
k_deg,m_ (1/day)	The degradation rate constant of mTFPI	(0.01, 100)	1.150	1.151 (1.146, 1.153)
k_deg,s_ (1/day)	The degradation rate constant of sTFPI-α	(0.025, 250) (Yuan et al., 2019)	75.50	75.52 (75.44, 75.56)
k_el,CM_ (1/day)	The elimination rate constant of MG1113/mTFPI complex	(0.01, 100)	0.006851	0.006848 (0.006579, 0.006945)
k_el,CS_ (1/day)	The elimination rate constant of MG1113/sTFPI-α complex	(0.00259, 25.9) (Kwak et al., 2021)	0.3094	0.3093 (0.3090, 0.3096)
k_el,MG1113_ (1/day)	The elimination rate constant of MG1113 at the central compartment	(0.0005359, 5.359) (Kwak et al., 2021)	0.4543	0.4542 (0.4540, 0.4550)
k_loss_ (1/day)	The elimination rate constant of MG1113 at the depot compartment	(0.01, 100)	3.149 × 10^−7^	4.419 × 10^−7^ (1.408 × 10^−12^, 1.141 × 10^−5^)
k_on_ (1/(nM × day))	The association rate constant of TFPI and MG1113	(0.01435, 143.5) (Kwak et al., 2021)	28.50	28.49 (28.42, 28.52)
mTFPI_base_ (nM)	The baseline concentration of mTFPI	(0.01, 100)	12.04	12.05 (12.03, 12.09)
sTFPI-α_base_ (nM)	The baseline concentration of sTFPI-α (Experimentally measured values)	(0.009768, 97.68)	0.9456	0.9455 (0.9454, 0.9457)

Briefly, the refined model incorporated MG1113 interactions with both sTFPI-α and mTFPI. The model assumed that MG1113 in the central compartment (C2_MG1113_) is eliminated through saturable binding to these targets and a first-order process defined by the elimination rate constant (k_el,MG1113_). The saturable binding of MG1113 to sTFPI-α and mTFPI was modeled using association (k_on_) and dissociation (k_off_) rate constants, where k_off_ was defined as the product of k_on_ and the equilibrium dissociation constant (K_D_). While the K_D_ value was adopted from the previous study (Kwak et al., 2020), the k_on_ was estimated as a fitted parameter in the refined model. The synthesis and degradation of sTFPI-α and mTFPI were set to follow zero- and first-order kinetics with rate constants: k_syn,s_ and k_deg,s_ for sTFPI-α, and k_syn,m_ and k_deg,m_ for mTFPI. Although sTFPI-α could theoretically originate from both *de novo* synthesis and the shedding of membrane-bound forms (Girard et al., 2012), it was not possible to distinguish these specific mechanisms with the current dataset. As a result, the refined model assumes a single zero-order production process (k_syn,s_) that accounts for all sources of sTFPI-α entering the systemic circulation. The resulting drug-target complexes, C2_CS_ and C2_CM_ were eliminated via first-order processes with rate constants, k_el,CS_ and k_el,CM_, respectively.

### 2.3 Parameter optimization by the cluster Gauss-Newton method (CGNM)

The CGNM enables the estimation of multiple solutions to non-linear least-squares problems and has been applied in physiologically-based PK (PBPK) and PD modeling (Aoki et al., 2022; Lee et al., 2023; Aoki and Sugiyama, 2024). CGNM identifies parameter sets through repeated estimations from randomly selected initial values, defined within a reported or user-specified range (typically 10^–2^ to 10^2^ of the base values; Table 1). Best-fit parameter sets are selected by minimizing the sum of squared residuals (SSR), calculated as:

$$SSR=\sum_{i=1}^{n}{\left(\log_{10}\left(y_{obs,i}\right)-\log_{10}\left(y_{pred,i}\right)\right)}^{2}$$

where *n* is the number of observations, and *y*_*obs,i*_ and *y*_*pred,i*_ denote the *i*th observed and model-predicted values. Unlike traditional optimization algorithms, such as the Levenberg-Marquardt method, CGNM does not assume a single optimal solution. Instead, CGNM evaluates parameter identifiability by identifying multiple parameter sets with similarly small SSR values, thereby distinguishing well-constrained parameters from those poorly informed by the data (Yoshikado et al., 2025). We leveraged this feature of CGNM to assess which model parameters were reliably estimated and which lacked sufficient support from the available experimental data.

The rxode2 package (version 3.0.1) was used to integrate the system of ODEs for a series of TMDD models, and CGNM was performed in RStudio (version 2024.04.1 Build 748) using the CGNM package (version 0.9.0). Mean values of the observed data were used for parameter optimization. For the model fitting, the number of initial parameter combinations (num_minimizersToFind) was set to 1,000, and the number of iterations (num_iteration) to 100, according to the user manual, with other settings left at default. The elbow method was used to detect a point of a sudden increase in the ordered SSR values, allowing for the selection of parameter sets with similarly small SSRs. Grubbs’ test (significance level = 0.05) was used to exclude outliers and obtain a final set of acceptable approximate minimizers. Parameter identifiability was assessed based on the approximated profile likelihood analysis as described previously (Aoki and Sugiyama, 2024).

### 2.4 Evaluation of the TMDD model

To assess the performance of the TMDD model, we compared the observed PK data with the corresponding model simulations. The predictive performance was quantitatively evaluated using the symmetric mean absolute percentage error (SMAPE), calculated based on the area under the curve to the last measurable concentration (AUC_last_) values from both the observed (AUC_obs_) and the simulated (AUC_sim_) profiles (Bussing and Dhaval, 2020). SMAPE can range from 0% to 200%, and is closer to 0% when the simulated and observed values are similar.

$$SMAPE=\frac{\left|{AUC}_{sim}-AUC_{obs}\right|}{\frac{1}{2}\left(\left|AUC_{sim}\right|+\left|AUC_{obs}\right|\right)}\times 100\%$$

### 2.5 PK study of MG1113 in rabbits

To evaluate the interspecies translatability of the refined TMDD model of MG1113 developed in monkeys, we obtained plasma MG1113 concentration-time profiles in rabbits and assessed whether the observed data were in good agreement with model-based predictions using parameters derived from allometric scaling. The animal study protocol was approved by the Institutional Animal Care and Use Committee of KPC (Gyeonggi-do, Republic of Korea). Male rabbits (2–3 months old; Orient Bio, Gyeonggi-do, Republic of Korea) received MG1113 via i.v. (2.75, 17.2, and 34.4 nmol/kg) or s.c. (17.2, 34.4, 68.8, and 137.6 nmol/kg) administration. Blood samples were drawn from the jugular vein at pre-defined time points post-administration (i.v. dosing, 0.00347, 0.0104, 0.0208, 0.0417, 0.167, 0.333, 0.5, 1, 2, 3, 4, 5, and 7 days; s.c. dosing, 0.0104, 0.0208, 0.0417, 0.167, 0.5, 1, 2, 3, 5, 14, and 21 days) into tubes containing 10% sodium citrate. Plasma was separated by centrifugation at 5,000 rpm for 20 min at 4 °C and stored at −75 °C ± 5 °C until quantification of MG1113 using a previously established ELISA (Kwak et al., 2021). Samples with signal intensities below the lowest calibration standard were treated as missing data (Beal, 2001).

### 2.6 Simulation of MG1113 and sTFPI-α profiles in rabbits and humans using parameters predicted by allometric scaling

Model parameters estimated from CGNM analysis of monkey PK/PD data were extrapolated to rabbits and humans using allometric scaling. For allometric scaling, we used representative body weights of each species (2.5 kg for rabbits, 70 kg for humans) based on the literature (Zhao et al., 2015). The allometric exponents used were 0.75 for clearance (CL_D_), −0.25 for rate constants (k_01_, k_02_, k_12_, k_deg,m_, k_deg,s_, k_el,CM_, k_el,CS_, k_el,MG1113_, and k_loss_), and 1.0 for volumes (V_2_ and V_3_) (Germovsek et al., 2021). The following formulas were applied:

$$CL_{i,species}={CL}_{i,monkey}\times {\left(\frac{Bodyweight_{species}}{Bodyweight_{monkey}}\right)}^{0.75}$$

$$k_{i,species}=k_{i,monkey}\times {\left(\frac{Bodyweight_{species}}{Bodyweight_{monkey}}\right)}^{-0.25}$$

$$V_{i,species}=V_{i,monkey}\times {\left(\frac{Bodyweight_{species}}{Bodyweight_{monkey}}\right)}^{1.0}$$

where *i* indicates each parameter.

The binding parameters of MG1113 to TFPI isoforms (e.g., K_D_ and k_on_) were assumed to be conserved across species based on the high sequence similarity of the K2 domain of TFPI (humans versus monkeys: 96% identity, 98% positive matches; humans versus rabbits: 92% identity, 98% positive matches). Baseline levels of sTFPI-α were set at 1.114 nM for rabbits and 2.3 nM for humans, based on published data (Kwak et al., 2020; Kwak et al., 2021). Baseline levels of sTFPI-α showed no reported difference between healthy adults and patients with hemophilia (Gu et al., 2015). Given that TFPI-β and TFPI-α are products of alternative splicing from the same TFPI gene (Maroney et al., 2010), the ratio of their estimated baseline levels in monkeys was used to establish the mTFPI baseline levels (mTFPI_base_) in humans and rabbits (Yuan et al., 2019). The following formula was applied:

$$mTFPI_{base,species}=sTFP{I‐\alpha }_{base,species}\times \frac{mTFPI_{base,monkey}}{sTFPI{‐\alpha }_{base,monkey}}$$

To assess the predictive accuracy of the refined model in rabbits, we compared the observed PK data with the corresponding model simulations. The predictive performance was quantitatively evaluated using the absolute average fold error (AAFE), calculated based on the observed and the predicted data (Saleh et al., 2024).

$$AAFE=10^{\frac{1}{n}\sum_{n}^{i}\left|\log_{10}\frac{y_{pred,i}}{y_{obs,i}}\right|}$$

where *n* is the number of observations, and *y*_*obs,i*_ and *y*_*pred,i*_ denote the *i*th observed and model-predicted values.

To assess the impact of parameters on simulation outcomes, a local sensitivity analysis was performed using both the refined model in the current study and the previously published model (Kwak et al., 2021). The AUC_last_ following a single administration was used as the primary model output representing systemic drug exposure. The sensitivity of the parameter was evaluated by calculating the percent change of AUC_last_:

$$\%change=\frac{{AUC}_{chg}-{AUC}_{sim}}{{AUC}_{sim}}\times 100$$

where AUC_sim_ is the AUC_last_ with rank 1 value of the parameter, and AUC_chg_ is the AUC after parameter adjustment. Simulations were performed using Berkeley Madonna (version 10.6.1, University of California, Berkeley, United States) and the resulting concentration-time profiles were visualized using GraphPad Prism (version 10.6.1, GraphPad Software, La Jolla, CA, United States).

To simulate the PK profiles of MG1113 in humans, we used dosing regimens from a phase I clinical trial (clinicaltrials.gov identifier: NCT03855696), which evaluated the safety, tolerability, and PK/PD profiles of MG1113 in healthy subjects and patients with hemophilia. Healthy subjects received the following MG1113 doses: s.c. doses of 0.5, 1.7, and 3.3 mg/kg, and i.v. dose of 3.3 mg/kg. Patients with hemophilia received s.c. MG1113 doses at 1.7 and 3.3 mg/kg.

## 3 Results

### 3.1 CGNM-based parameter optimization for the refined TMDD model of MG1113: fitting to the data from monkeys

The refined TMDD model for MG1113 and sTFPI-α included 15 estimated parameters (Figure 1; Table 1). Of the final 1,000 parameter sets generated through iterative CGNM optimization, 136 sets met the acceptance criterion (SSR ≤6.34) and were used for subsequent analyses (Supplementary Figure S3). Thirteen out of the fifteen parameters exhibited narrow distributions among the accepted sets (Table 1; Supplementary Figure S4). The rank 1 value of V_2_ (0.1112 L) was comparable to the reported monkey plasma volume (0.157 L) (Zhao et al., 2015). The rank 1 value of V_3_ (0.1346 L) was comparable to the values of the peripheral compartment volume reported for other anti-TFPI antibodies in cynomolgus monkeys (0.1155 L and 0.2121 L) (Agerso et al., 2014; Gu et al., 2017). The rank 1 value of sTFPI-α_base_ (0.9456 nM) closely matched the experimentally measured value (0.9768 nM). The rank 1 value of k_deg,s_ (75.5/day) was comparable to that reported for another anti-TFPI antibody in cynomolgus monkeys (98.6/day) (Gu et al., 2017). Five parameters (k_02_, k_el,CM_, k_el,CS_, k_loss_, and k_on_) were deemed non-identifiable (Supplementary Table S1; Supplementary Figure S5). The small values of k_loss_ and k_02_ suggested that MG1113, administered subcutaneously, is almost completely absorbed, albeit with a delay. The k_on_ parameter was deemed non-identifiable due to two local minima in the approximate profile likelihood plots (Supplementary Figure S5). The subsequent analysis was conducted using the first minimum value, which was included in the range of accepted parameter sets (Table 1).

Fixing parameters k_02_ and k_loss_ to zero (reflecting their minute estimated values) during the CGNM analysis resulted in negligible changes to the remaining parameter estimates and the min SSR (Supplementary Table S2). This indicates that the omission of these parameters would not compromise model performance. Nevertheless, they were retained to maintain a general model applicable to other antibody therapeutics.

**FIGURE 2:**
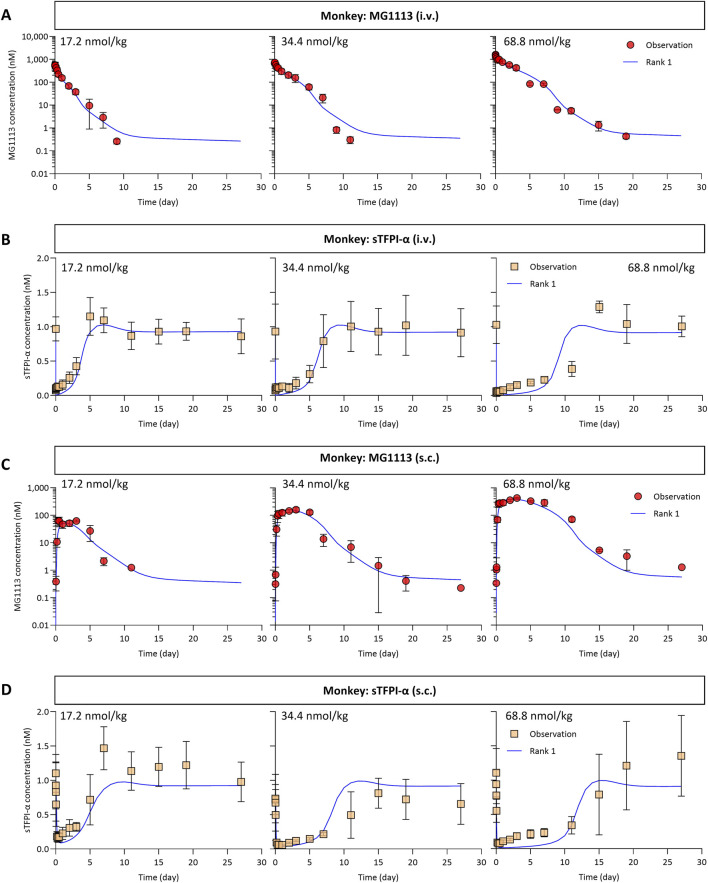
The plasma concentration-time profiles of MG1113 **(A,C)** and sTFPI-α **(B,D)** from cynomolgus monkeys using the optimized parameters obtained via the CGNM-based analyses. The lines show the model-fitted profiles (using the 136 accepted parameter sets) which nearly overlap. The symbols represent the observed data obtained after a single i.v. or s.c. administration at 17.2, 34.4, and 68.8 nmol/kg. Each data point represents the mean concentration along with the corresponding standard deviation (*n* = 1-3 per group). sTFPI-α, soluble tissue factor pathway inhibitor alpha.

The 136 simulated plasma concentration-time profiles of MG1113, generated using the accepted parameter sets, were nearly superimposable and closely aligned with the observed data (Figure 2A for i.v. dosing; Figure 2C for s.c. dosing). To quantitatively evaluate the predictive performance of the TMDD model in monkeys, we calculated the SMAPE based on the AUC_last_ from both the observed and the simulated profiles. The model-predicted AUC_last_ values were in good agreement with the observed data, with the SMAPE ranging from 2.0% to 30.2% (Table 2). Similarly, the simulated sTFPI-α profiles were also nearly superimposable and reasonably aligned with the observed data (Figures 2B,D). The model-predicted AUC_last_ values were in good agreement with the observed data, with the SMAPE ranging from 3.2% to 28.8% (Table 2). Overall, the agreement between model-based predictions and observed data was reasonably good, as supported by goodness-of-fit plots (Supplementary Figure S6).

**TABLE 2:** Comparison of the observed and model-predicted AUC_last_ values for MG1113 and sTFPI-α in monkeys that received i.v. or s.c. administration of MG1113.

Article	Route	Dose (nmol/kg)	AUC_last_ (day × nM)		SMAPE (%)
			Observed	Predicted	
MG1113	i.v	17.2	510.4	459.1	10.6
		34.4	1199.8	1243.2	3.6
		68.8	2918.5	3137.1	7.2
	s.c	17.2	274.6	202.6	30.2
		34.4	875.4	858.4	2.0
		68.8	3233.6	2624.5	20.8
sTFPI-α	i.v	17.2	22.9	22.2	3.2
		34.4	21.1	20.0	5.3
		68.8	18.5	17.5	5.3
	s.c	17.2	27.6	21.1	26.3
		34.4	13.6	18.1	28.8
		68.8	19.0	15.0	23.4

SMAPE, symmetric mean absolute percentage error; AUC_last_, the area under the curve to the last measurable point; sTFPI-α, soluble tissue factor pathway inhibitor alpha; i.v., intravenous; s.c., subcutaneous.

### 3.2 Simulation of plasma MG1113 and sTFPI-α profiles in rabbits

**FIGURE 3:**
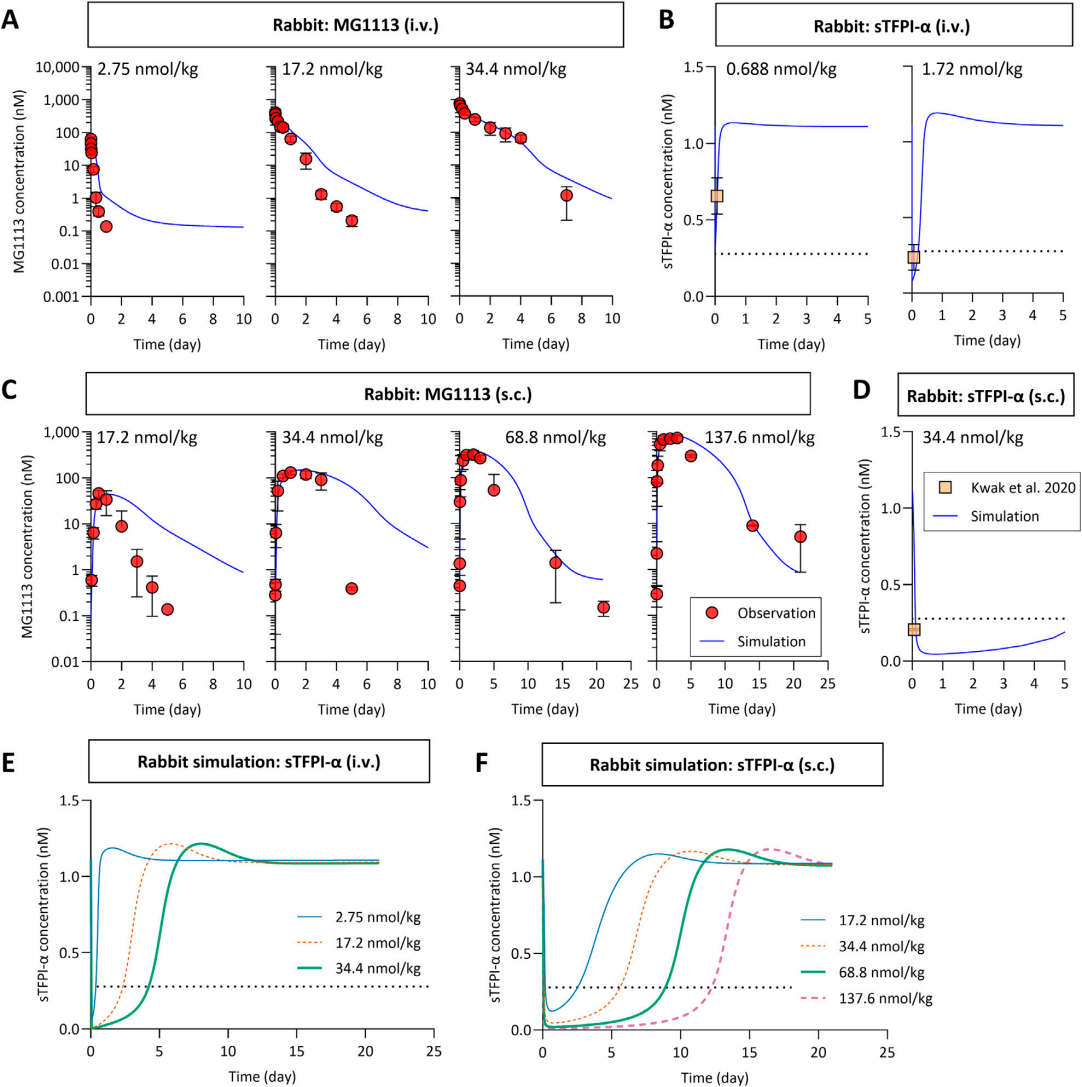
The simulated plasma concentration-time profiles of MG1113 **(A,C)** and sTFPI-α **(B,D–F)** in rabbits after a single i.v. or s.c. administration. **(B,D)** The mean observed concentrations of sTFPI-α at 90 min post-dosing were obtained from Kwak et al. (2020). **(B,D–F)** The dotted horizontal line indicates 25% of the baseline level of sTFPI-α. The symbols represent the mean observed concentrations along with the corresponding standard deviation (*n* = 2–10 per group). sTFPI-α, soluble tissue factor pathway inhibitor alpha.

We utilized the refined TMDD model and allometrically scaled parameters based on the rank 1 values to simulate the plasma concentration-time profiles of MG1113 and sTFPI-α in rabbits (Table 3). Simulations were performed across various dosing conditions, following either single i.v. or s.c. administration. The simulated MG1113 profiles showed good agreement with observed values in the high-dose groups (i.v. dose of 34.4 nmol/kg and s.c. doses of 68.8 and 137.6 nmol/kg) with AAFE values ranging from 1.5 to 1.9 (Figures 3A,C). However, in the low-dose groups (i.v. doses of 2.75 and 17.2 nmol/kg and s.c. doses of 17.2 and 34.4 nmol/kg), the model tended to overpredict MG1113 concentrations with AAFE values ranging from 2.8 to 4.2, not capturing the rapid decline of MG1113 (Figures 3A,C).

**TABLE 3:** Predicted rabbit and human parameters by allometric scaling with rank 1 values of each parameter.

Parameter (unit)	Monkey	Rabbit	Human
Body weight (kg)	3.5 (Zhao et al., 2015)	2.5 (Zhao et al., 2015)	70.0 (Zhao et al., 2015)
K_D_ (nM)	0.04665	0.04665	0.04665
CL_D_ (L/day)	0.1454	0.1130	1.375
V_2_ (L)	0.1112	0.07944	2.224
V_3_ (L)	0.1346	0.09615	2.692
k_01_ (1/day)	20.32	22.10	9.609
k_02_ (1/day)	8.859 × 10^−8^	9.637 × 10^−8^	4.189 × 10^−8^
k_12_ (1/day)	0.4033	0.4387	0.1907
k_deg,m_ (1/day)	1.150	1.251	0.5440
k_deg,s_ (1/day)	75.50	82.13	35.70
k_el,CM_ (1/day)	0.006851	0.007452	0.003240
k_el,CS_ (1/day)	0.3094	0.3366	0.1463
k_el,MG1113_ (1/day)	0.4543	0.4942	0.2148
k_loss_ (1/day)	3.149 × 10^−7^	3.425 × 10^−7^	1.489 × 10^−7^
k_on_ (1/(nM × day))	28.50	28.50	28.50
mTFPI_base_ (nM)	12.04	14.19	29.11
sTFPI-α_base_ (nM)	0.9456	1.114 (Kwak et al., 2020)	2.3 (Kwak et al., 2021)

Unfortunately, the sTFPI-α profiles over time were unavailable from the same rabbits that received the i.v. and s.c. doses of MG1113. Our previous study (an MG1113 efficacy study in the rabbit model of hemophilia A) measured plasma sTFPI-α levels 90 min post-dose (i.v. doses of 0.1 and 0.25 mg/kg, equivalent to 0.688 and 1.72 nmol/kg; s.c. dose of 5.0 mg/kg, equivalent to 34.4 nmol/kg) (Kwak et al., 2020). When the observed values at 90 min post-dose were compared to model-predicted sTFPI-α levels, good agreement was observed (Figures 3B,D). These results support the overall translational applicability of the monkey-based TMDD model to rabbits, although further refinement may be needed for low-dose or s.c. administration.

### 3.3 Simulation of MG1113 and sTFPI-α profiles in humans

**FIGURE 4:**
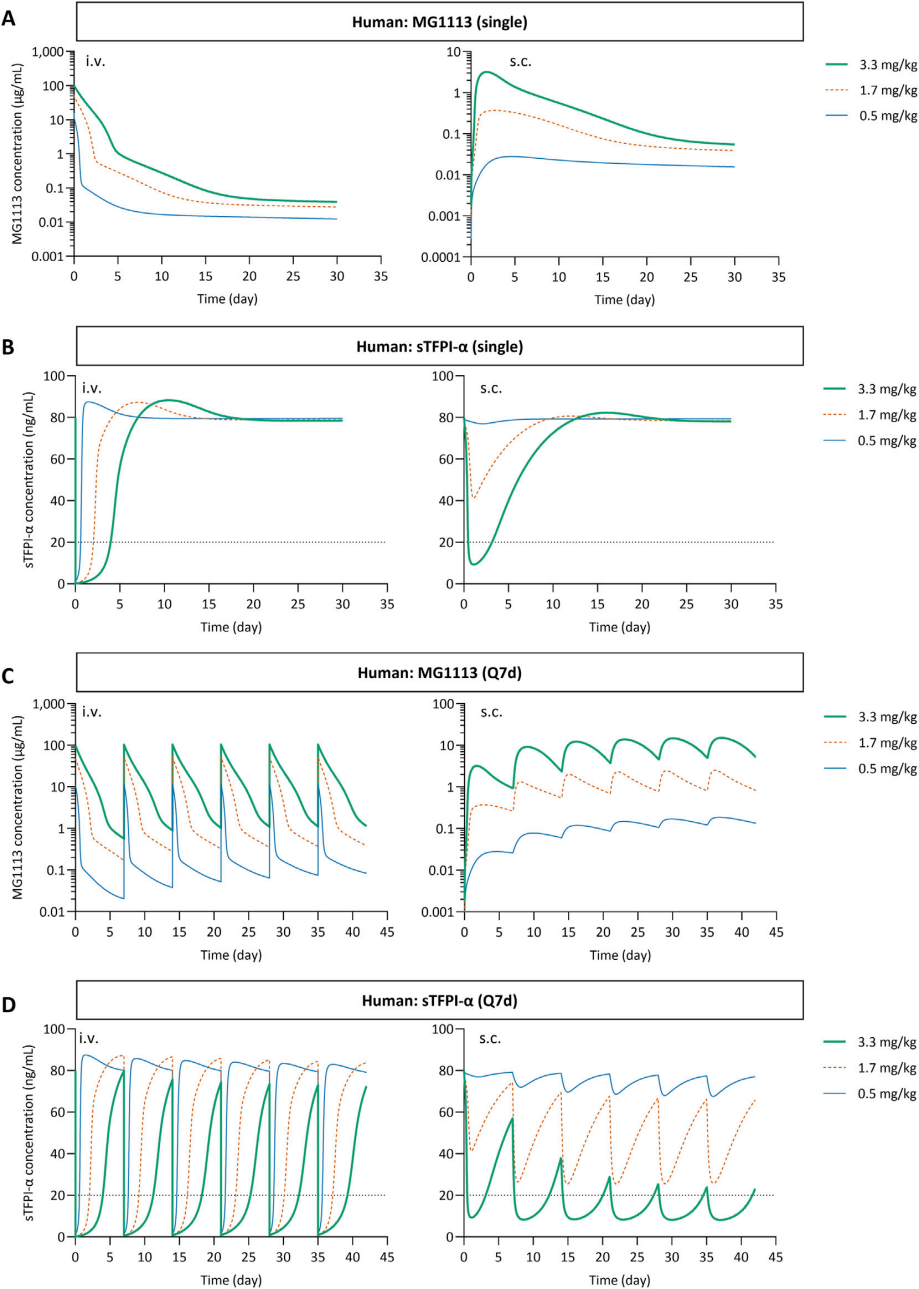
The simulated plasma concentration-time profiles of MG1113 and sTFPI-α in humans after single **(A,B)** or multiple **(C,D)** administration via i.v. or s.c. routes. **(B,D)** The dotted horizontal line indicates 25% of the baseline level of sTFPI-α. sTFPI-α, soluble tissue factor pathway inhibitor alpha; Q7d, every 7 days.

The refined TMDD model, along with allometrically scaled parameters based on the rank 1 values, was used to predict the plasma concentration-time profiles of MG1113 and sTFPI-α in humans (Table 3). Simulations were conducted under the phase I dosing regimens (0.5, 1.7, and 3.3 mg/kg, equivalent to 3.44, 11.7, and 22.7 nmol/kg) for both single and multiple (weekly, Q7d) i.v. and s.c. administrations (Figure 4). To compare the systemic exposure of MG1113 across the dose ranges, the dose-normalized AUC values were calculated for i.v. and s.c. dosing. The results indicated a more-than-dose-proportional increase, with a greater extent in s.c. groups than i.v. groups (Supplementary Table S3).

Single i.v. administration of MG1113 reduced sTFPI-α to less than 25% of baseline across all doses, while s.c. dosing required 3.3 mg/kg to achieve this outcome (Figure 4B). At 3.3 mg/kg, the sTFPI-α level remained suppressed below 25% of baseline for approximately 3 days. Rebound of sTFPI-α was predicted after i.v. dosing, more prominently than s.c. dosing (Figure 4B). After weekly i.v. administration of MG1113, sTFPI-α levels rebounded above baseline before the next dose at 0.5 and 1.7 mg/kg (3.44 and 11.7 nmol/kg) (Figure 4D). The rebound phenomenon was less pronounced with s.c. dosing and weekly dosing of 3.3 mg/kg maintained sTFPI-α levels below 25% of baseline for the majority of the dosing interval (Figure 4D).

**FIGURE 5:**
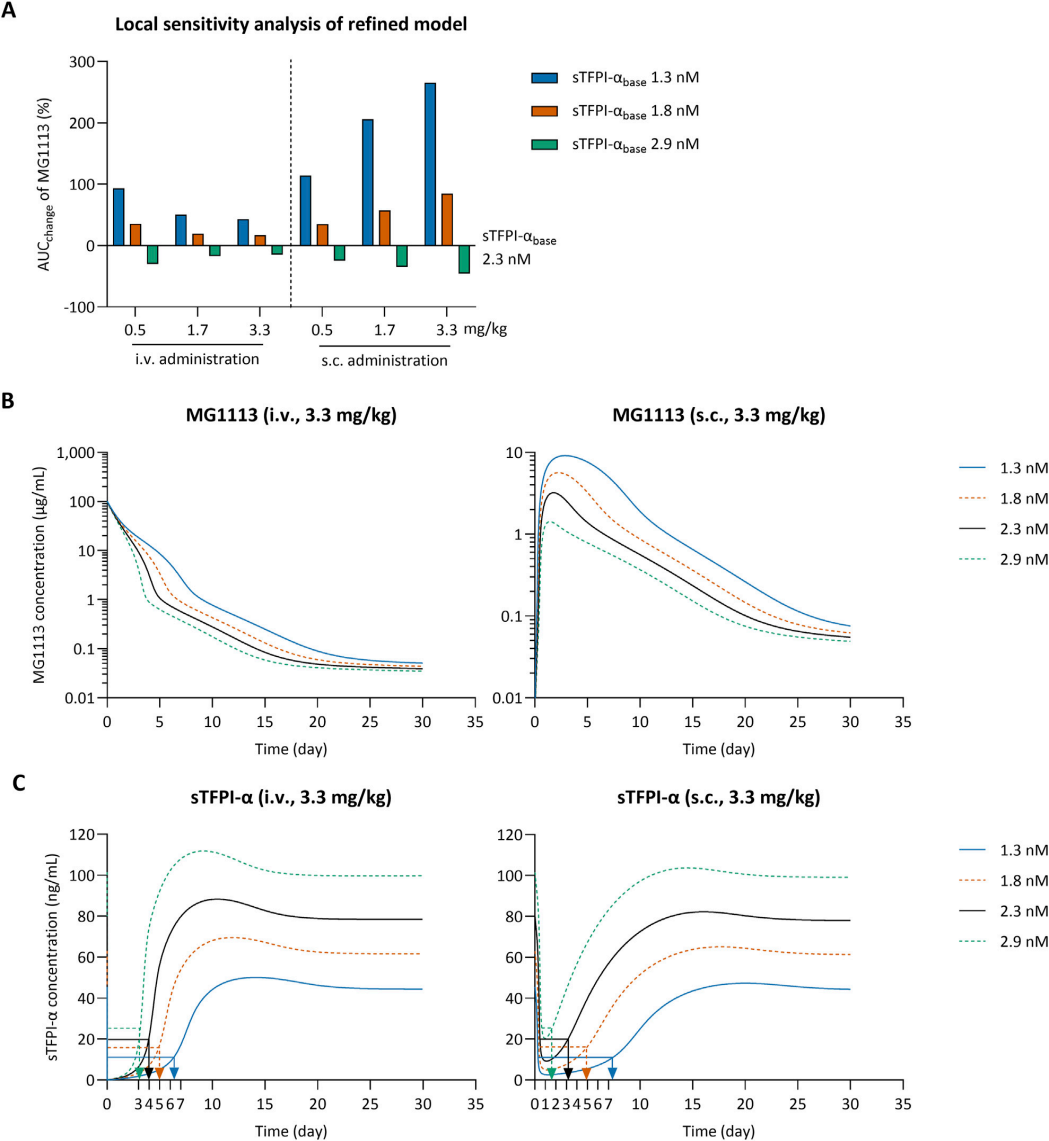
The results of the local sensitivity analysis of the baseline concentration of soluble tissue factor pathway inhibitor alpha (sTFPI-α_base_) of the refined TMDD model for MG1113 in human prediction. **(A)** Relative changes in the predicted AUC_0–30days_ of MG1113 in humans, in response to changes in sTFPI-α_base_. The simulated plasma profiles of MG1113 **(B)** and sTFPI-α **(C)** in humans after i.v. or s.c. dosing of 3.3 mg/kg (22.7 nmol/kg), using the changed values of sTFPI-α_base_. Each arrowed lines indicate the duration of sTFPI-α suppression below 25% of baseline for its corresponding condition.

The 95% prediction interval of sTFPI-α_base_ in human plasma was estimated to be 1.3–2.9 nM based on the previous study (Dahm et al., 2003). A local sensitivity analysis was conducted to assess its impact on the predicted MG1113 and sTFPI-α profiles in humans receiving 3.3 mg/kg of MG1113 via either i.v. or s.c. route. To maintain the ratio between the sTFPI-α_base_ and mTFPI_base_, the mTFPI_base_ value was adjusted accordingly and subsequently applied in the local sensitivity analysis (mTFPI_base_ = 12.7 × sTFPI-α_base_). The results showed that the MG1113 exposure (e.g., the % change in AUC_0–30days_) was more sensitive to sTFPI-α_base_ variation in the s.c. route than the i.v. route. As sTFPI-α_base_ increased from 2.3 nM to 2.9 nM, the AUC_0–30days_ of MG1113 decreased (Figures 5A,B), and the duration for which sTFPI-α levels were maintained at less than 25% was decreased (Figure 5C). These predictions are consistent with the increase in the target binding capacity as sTFPI-α_base_ values increase. At a dose of 3.3 mg/kg, when sTFPI-α_base_ was reduced to 1.3 nM, MG1113 could suppress sTFPI-α to below 25% of baseline for approximately 6–7 days regardless of the dosing route (Figure 5C). These findings support considering individual baseline sTFPI-α levels when optimizing MG1113 dosing in patients with hemophilia. When a similar local sensitivity analysis was performed using the previous model (Kwak et al., 2021), the changes in sTFPI-α_base_ showed only a minimal influence on the AUC_0–30days_ of MG1113 (Supplementary Figure S9).

## 4 Discussion

Anti-TFPI antibodies have gained attention as novel therapeutics for hemophilia, with recent FDA approvals. As MG1113 has shown potent neutralizing effects on both soluble and membrane-associated TFPIs (Kwak et al., 2020), the current study refined a previous TMDD model of MG1113, which focused solely on sTFPI-α. This study aimed to improve physiological plausibility and facilitate interspecies prediction by incorporating mTFPI kinetics (Figure 1). The refined model showed overall improved performance compared to the previous model when fitted to monkey data (Supplementary Table S4). The refined model was externally validated in rabbits (Figure 3) and extended to humans (Figures 4, 5), capturing the nonlinear PK/PD characteristics of MG1113 across species.

Refinement for the MG1113 PK/PD model was necessary to address several limitations of the previous TMDD model. In TMDD kinetics, the abundance of drug-interacting targets is a key determinant of nonlinear behavior. However, the previous model accounted only for the sTFPI-α, which constitutes less than 10% of the total TFPI pool, and therefore did not fully capture the underlying physiology of TFPI. In addition, the previous model described the rebound of sTFPI-α utilizing an empirical feedback compartment. Because the parameters governing this feedback process lacked clear biological interpretation, we sought to replace it with a mechanistic framework capable of providing biological insight into the rebound phenomenon and allowing reasonable interspecies extrapolation. Furthermore, a transit compartment for s.c. absorption of MG1113 was incorporated to better represent the delayed systemic absorption typically mediated via the lymphatic route for large molecules (Sanchez-Felix et al., 2020; Viola et al., 2018).

To enhance the biological interpretability of the model and allow for a more reasonable interspecies scaling, the refined model incorporated the saturable binding of MG1113 to mTFPI, which represents the major fraction of total TFPIs. Incorporating mTFPI kinetics enables the model to capture the full binding capacity and turnover, providing a more mechanistic description of MG1113 disposition across species. As such, a previous model for concizumab incorporated both mTFPI and sTFPI interactions, demonstrating that mTFPI-mediated endocytosis plays a major role in the nonlinear PK behavior of anti-TFPI antibodies (Yuan et al., 2019). Previous TMDD models for anti-TFPI antibody therapeutics often used the Michaelis-Menten (MM) approximation to describe the interaction with mTFPI (Agerso et al., 2014; Parng et al., 2018). However, this simplification may not be appropriate for high-affinity antibodies such as MG1113 (K_D_ = 0.04665 nM). The MM approximation may be applicable when the total target concentration (R_tot_) is much lower than the dissociation constant (K_D_) (Yan et al., 2010; Straube, 2025). In contrast, the measured sTFPI-α level in monkeys (0.9768 nM), which represents only a fraction of R_tot_, exceeds the K_D_ of MG1113 (0.04665 nM) by more than 20-fold, and the concentration of mTFPI is expected to be even higher (e.g., K_D_ ≪ R_tot_) (Girard et al., 2012). This quantitative disparity violates the necessary assumption of the MM approximation. Therefore, our refined model explicitly represented the mTFPI-MG1113 interaction using the association and dissociation rate constants (Figure 1).

To simplify the model, k_el,CM_ may be set equal to k_deg,m_, which assumes the concentration of the mTFPI pool is constant. However, assuming a constant mTFPI pool may not reflect the actual physiological dynamics following MG1113 administration. There is no experimental evidence to guarantee that the mTFPI pool remains constant in the presence of MG1113. Furthermore, antibody binding can alter the internalization kinetics of membrane-bound targets, depending on the epitope and binding affinity (Opalinski et al., 2018). Considering these, therefore, we chose to include the two separate parameters, k_el,CM_ and k_deg,m_, in the model.

sTFPI-α has been widely used as a plasma biomarker for monitoring treatment efficacy, with levels below 25% of baseline indicating a positive response (Chowdary et al., 2015; Eichler et al., 2018). In monkeys receiving MG1113, there was a noticeable rebound in plasma sTFPI-α levels (Figure 2; Supplementary Figure S2), which may increase bleeding risk. Thus, it is important to enhance our mechanistic understanding by adequately capturing the rebound behavior in the model. The previous model (Kwak et al., 2021) described the sTFPI-α rebound using a separate feedback compartment, which offered limited biological insights. In the current study, the refined model adopted a mechanism-based approach. Theoretical studies (Aston et al., 2014; Aston et al., 2017) suggested that a rebound can occur when the internalization rate of the drug-target complex (k_el,CS_) is slower than both the elimination rate of free drug (k_el,MG1113_) and the degradation rate of free target (k_deg,s_). Accordingly, the refined model addressed the challenges of interspecies translation by reflecting the sTFPI-α rebound through the kinetic relationship. The CGNM analysis estimated that k_el,MG1113_ (0.4543/day) and k_deg,s_ (75.50/day) were greater than k_el,CS_ (0.3094/day) (Table 1). Consistent with this kinetic relationship, the model successfully captured the sTFPI-α rebound in the observed data (Figure 2).

The estimated k_deg,s_ (75.5/day) may appear high, implying a rapid turnover of sTFPI-α. However, this estimate is consistent with the previous finding for another anti-TFPI antibody (e.g., 98.6/day) (Gu et al., 2017). Physiologically, this rapid turnover should be interpreted in the context of the steady-state relationship: k_syn,s_ = k_deg,s_ × sTFPI-α_base_. A high degradation rate necessitates a correspondingly high k_syn,s_ to maintain baseline levels. In our refined model, k_syn,s_ represents a lumped parameter that may encompass not only *de novo* synthesis but also the constitutive release and shedding of sTFPI-α from the platelets and vascular endothelium.

Following s.c. administration of MG1113 in monkeys, the time to reach the maximum concentration (T_max_) ranged from 32 to 72 h (Kwak et al., 2021), similar to other monoclonal antibodies administered in the same species (Viola et al., 2018). In this study, a transit compartment was adopted to describe the absorption kinetics. In the refined model, the k_loss_ was negligible, supporting nearly complete absorption of s.c. doses. Furthermore, the k_02_ was found to be non-identifiable with minute values (close to zero), suggesting that the dominant absorption route may involve the passage through the transit compartment rather than direct absorption from the injection depot into the plasma. Given the molecular weight of MG1113 (145.36 kDa), the lymphatic route is expected to serve as the primary absorption mechanism following s.c. administration (Sanchez-Felix et al., 2020; Viola et al., 2018).

When the refined TMDD model of MG1113 was used for interspecies scaling, the simulated PK profiles of MG1113 in rabbits tended to slightly overpredict the observed values, particularly in the low-dose group, suggesting incomplete target occupancy (Figure 3). Overall, the nonlinear PK profiles of MG1113 in rabbits could not have been adequately described without sufficient target abundance. The local sensitivity analysis (data not shown) indicated that increasing either k_deg,m_ or mTFPI_base_ improved the model fit for the low-dose group. However, this adjustment caused instability in the fitting of the high-dose group, so we decided to maintain the current parameter values. Although further verifications are warranted, these findings highlight the importance of accurately characterizing the baseline level and turnover rate of TFPI in rabbits for reliable interspecies prediction of MG1113 PK profiles.

Due to the unavailability of MG1113 clinical trial data, we could not validate the simulated profiles of MG1113 and sTFPI-α in humans. However, the present modeling effort offers predictions of clinical outcomes. Our simulations predict that weekly s.c. administration mitigated the rebound, maintaining sTFPI-α levels below 25% of baseline at the 3.3 mg/kg dose in steady-state conditions (Figure 4D). This suggests a potential benefit of s.c. administration in managing sTFPI-α levels and associated bleeding risks. When comparing the predicted human profiles from the previous model (Kwak et al., 2021) and the current refined TMDD model, substantial differences were noted (Supplementary Figure S7–S9). In particular, the magnitude of influence of sTFPI-α_base_ and the doses on the systemic exposure of MG1113 varied considerably between the models (Supplementary Figure S9). Overall, the refined TMDD model, which incorporates biologically relevant target interactions, may be better suited to explain interindividual variability and personalize dosing regimens based on sTFPI-α levels (detailed in Supplementary Material).

Although the TMDD model for MG1113 was well established, a discrepancy was noted in the estimated kinetic parameter. Specifically, the estimated kon value was 28.5/(nM × day), which is approximately 10-fold lower than the previously reported value (Kwak et al., 2020). This discrepancy may result from the inherent differences between *in vitro* and *in vivo* conditions. *In vitro* binding assays are conducted in a controlled environment that includes only sTFPI-α and MG1113, whereas *in vivo* kinetics are likely influenced by additional proteins, membrane interactions, and various physiological factors (Tang and Cao, 2021). Despite the difference in k_on_, the dissociation constant (K_D_) was comparable to the previously reported value, indicating that these biological factors may proportionally influence both the association and dissociation rates. Therefore, we used the K_D_ from *in vitro* measurements, which aligns with common practices in antibody PK/PD modeling. This modeling strategy offers a practical framework for investigating antibody-target interactions under physiological conditions (Tang and Cao, 2021; Kastritis et al., 2011). Alternatively, the quasi-steady-state (QSS) or the quasi-equilibrium (QE) approximations could be considered (Gu et al., 2017; Parng et al., 2018). However, because these methods assume rapid equilibrium, they cannot adequately describe the early steep decline phase (Dua et al., 2015). Our dataset includes intensive early-time sampling (5, 15, 30, and 60 min) that could capture this rapid initial decline (Figure 2). Therefore, QSS or QE approximations were deemed inappropriate, and the full TMDD model structure was employed in this study.

In conclusion, the refined TMDD model offers enhanced physiological plausibility by incorporating key aspects of TFPI biology and antibody absorption kinetics. This model may serve as a valuable framework for future validation and for improving the predictability of MG1113 PK profiles in patients with hemophilia.

## Data Availability

The data analyzed in this study is subject to the following licenses/restrictions: The data that support the findings of this study are available from the first author upon reasonable request. Requests to access these datasets should be directed to Heechun Kwak, hcmarine@gccorp.com.
