# Supplementary material for: Refined target-mediated drug disposition modeling of the anti-tissue factor pathway inhibitor antibody MG1113 in cynomolgus monkeys and rabbits
Source: Front Pharmacol. 2026 Jan 7;16:1745702. doi: 10.3389/fphar.2025.1745702 (PMC12819659; doi:10.3389/fphar.2025.1745702)
Supplement: Supplementary file 1 [file DataSheet1.pdf]

Supplementary Material

Refined target-mediated drug disposition modeling of the anti-tissue factor pathway inhibitor antibody MG1113 in cynomolgus monkeys and rabbits

1 Supplementary Results

Comparison of human simulation results using previous and refined models

We compared human simulation results between the previous (1) and refined TMDD models. Following a single intravenous (i.v.) administration, the refined model predicted a steeper initial decline and slower terminal elimination of MG1113 compared to the previous model (Figure S7A). These differences likely reflect the larger capacity of the target pool in the refined model, which includes sTFPI-α and mTFPI. A similar trend was observed after a single subcutaneous (s.c.) administration (Figure S7C), with the refined model predicting lower C_{max} values. In terms of sTFPI-α profiles, the previous model showed a more pronounced rebound effect and a longer duration below 25% of baseline (Figure S7B and D). With repeated weekly dosing, differences in the plasma concentration-time profiles of MG1113 and sTFPI-α persisted between the two models, largely attributable to differences in target pool assumptions (Figure S8). However, at steady state following weekly s.c. administration of 3.3 mg/kg, both models yielded similar predictions (Figure S8C, D). This suggests that this s.c. dosing regimen may mitigate the target rebound effect. At lower doses and during non-steady state conditions, the pharmacokinetic (PK) and pharmacodynamic (PD) profiles of MG1113 and sTFPI-α could still be influenced by the extent of the target rebound effect.

Given the interindividual variability in baseline sTFPI-α levels, we conducted a sensitivity analysis to evaluate the impact of these differences on the model predictions. Using the area under the curve from 0 to 30 days ($AUC_{0\text{-}30days}$) of MG1113 as the metric, we found that the refined model was more sensitive to changes in sTFPI-α baseline levels, particularly under s.c. dosing (Figure S9A). Furthermore, the dose-normalized $AUC_{0\text{-}30days}$ varied more markedly with dose in the refined model, more prominently in the s.c. route (Figure S9B).

2 Appendix

Model Assumptions and Justifications

① Although sTFPI-α could theoretically originate from both *de novo* synthesis and the shedding of membrane-bound forms (2), it was not possible to distinguish these specific mechanisms with the current dataset. As a result, the refined model assumes a single zero-order production process ($k_{syn,s}$) that accounts for all sources of sTFPI-α entering the systemic circulation to prevent over-parameterization.

② The interaction between MG1113 and mTFPI was described using association and dissociation rate constants, since the Michaelis-Menten approximation is inappropriate for high-affinity antibodies like MG1113 (K_D = 0.04665 nM) (3, 4).

③ The interaction between MG1113 and sTFPI-α was described using association and dissociation rate constants, since the quasi-steady-state and the quasi-equilibrium approximations are inappropriate to capture the rapid initial decline (5).

④ Although sTFPI-α can distribute into the interstitial space, the refined model confines the description of drug-target interactions to the central compartment. Distinguishing peripheral from central target elimination was unfeasible, relying solely on plasma concentration data. Consequently, peripheral pathways were excluded to prevent model over-parameterization and ensure parameter identifiability without compromising predictive accuracy.

⑤ The neonatal fragment crystallizable receptor (FcRn)-mediated recycling was modeled using a single first-order elimination rate constant ($k_{el,MG1113}$) rather than an endosomal compartment. This parsimonious approach is justified because the rate-limiting step is the relatively slow nonspecific pinocytic uptake (~1.09 /h), whereas intracellular FcRn binding is rapid (~39,441 /h) and occurs under non-saturating conditions (concentration < K_D ≈ 101 nM) (6, 7). Consequently, the overall process follows linear first-order kinetics, allowing the lumped parameter to robustly describe the data while ensuring parameter identifiability.

⑥ The target binding-related parameters (e.g., K_D, k_{on}, k_{off}) of MG1113 were assumed to be conserved across species (human, monkey, and rabbit) and across the target forms (sTFPI-α and mTFPI). This assumption was based on the high sequence similarity of the K2 domain of the TFPIs across these species.

⑦ Since TFPI-β and TFPI-α are derived from the same TFPI gene through alternative splicing (8), the $mTFPI_{base}$ in humans and rabbits was established by applying the estimated ratio of $mTFPI_{base}$ to $sTFPI\text{-}\alpha_{base}$ in monkeys (9).

$$mTFPI_{base,species} = sTFPI\text{-}\alpha_{base,species} \times \frac{mTFPI_{base,monkey}}{sTFPI\text{-}\alpha_{base,monkey}} = 12.7 \times sTFPI\text{-}\alpha_{base,species}$$

⑧ Following subcutaneous administration, MG1113 was assumed to transfer to a transit compartment before entering the plasma compartment. This was supported by the molecular weight of MG1113 (145.36 kDa), which renders the lymphatic route as the primary mechanism for absorption (10, 11).

3 Supplementary Figures and Tables

3.1 Supplementary Figures

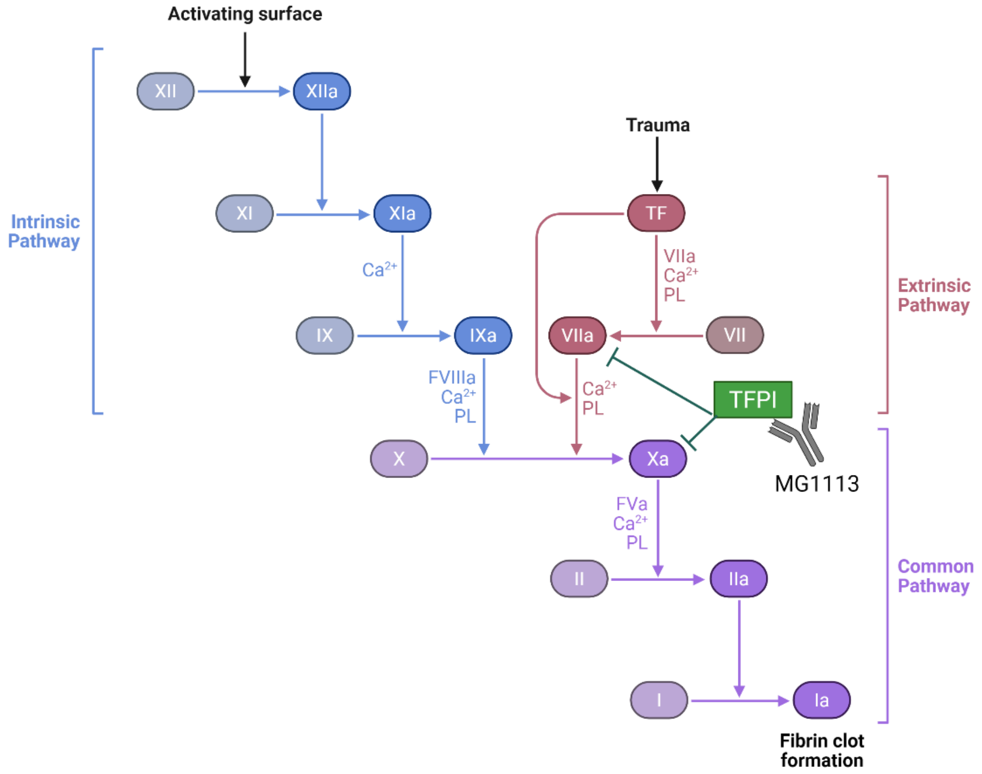

Figure S1. The mechanism of action of MG1113 in promoting hemostasis in hemophilia. In hemophilia, the intrinsic pathway is impaired due to a deficiency in Factor VIII or IX, leading to inadequate thrombin generation. Tissue Factor Pathway Inhibitor (TFPI) acts as a regulator, blocking both the extrinsic FXase (TF-FVIIa complex) and activated Factor X (FXa). MG1113 is a monoclonal antibody that specifically binds and neutralizes TFPI. By inhibiting TFPI, MG1113 promotes the extrinsic pathway-mediated activation of FX, thereby enhancing thrombin generation and restoring hemostasis to compensate for the intrinsic pathway defect. PL, phospholipid (Created in BioRender. Kwak, H. (2025) https://BioRender.com/oshr8px)

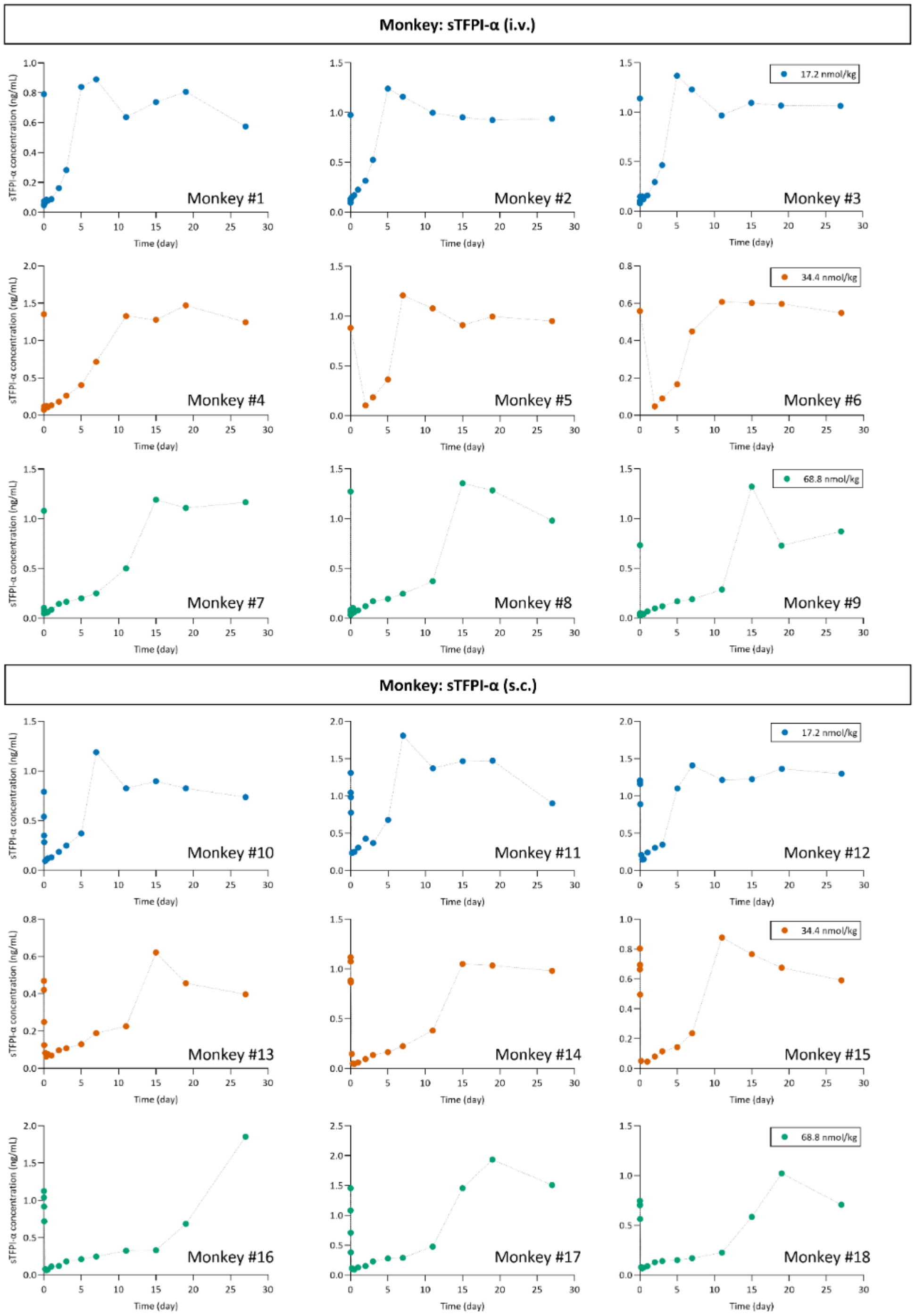

Figure S2. The plasma concentration-time profiles of sTFPI-α from individual cynomolgus monkeys. The symbols represent the observed data obtained after a single i.v. or s.c. administration at 17.2, 34.4, and 68.8 nmol/kg. sTFPI-α, soluble tissue factor pathway inhibitor alpha.

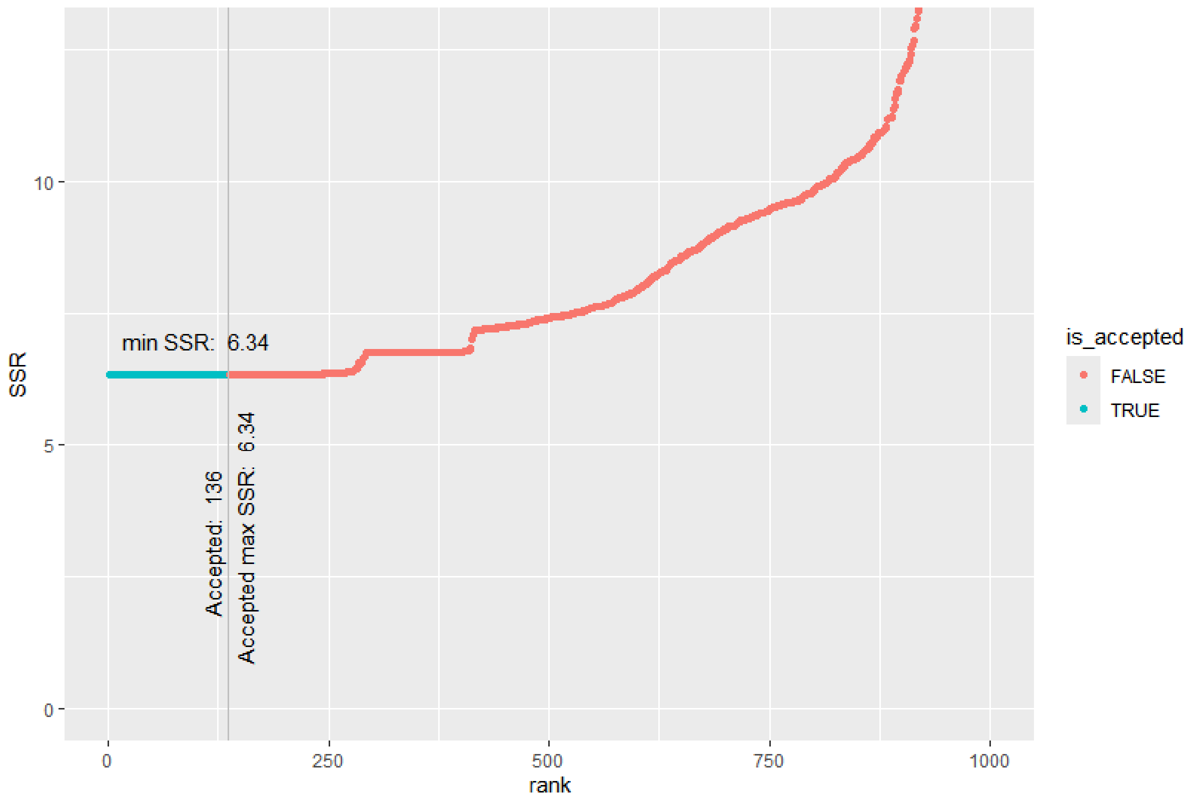

Figure S3. Plot showing the sum of squared residuals (SSR) values for parameter sets estimated by the Cluster Gauss-Newton method (CGNM). The SSR values of 1,000 parameter sets were plotted in ascending order. Green dots indicate the 136 parameter sets that met the acceptance criteria (SSR values less than 6.34), whereas red dots represent those that did not.

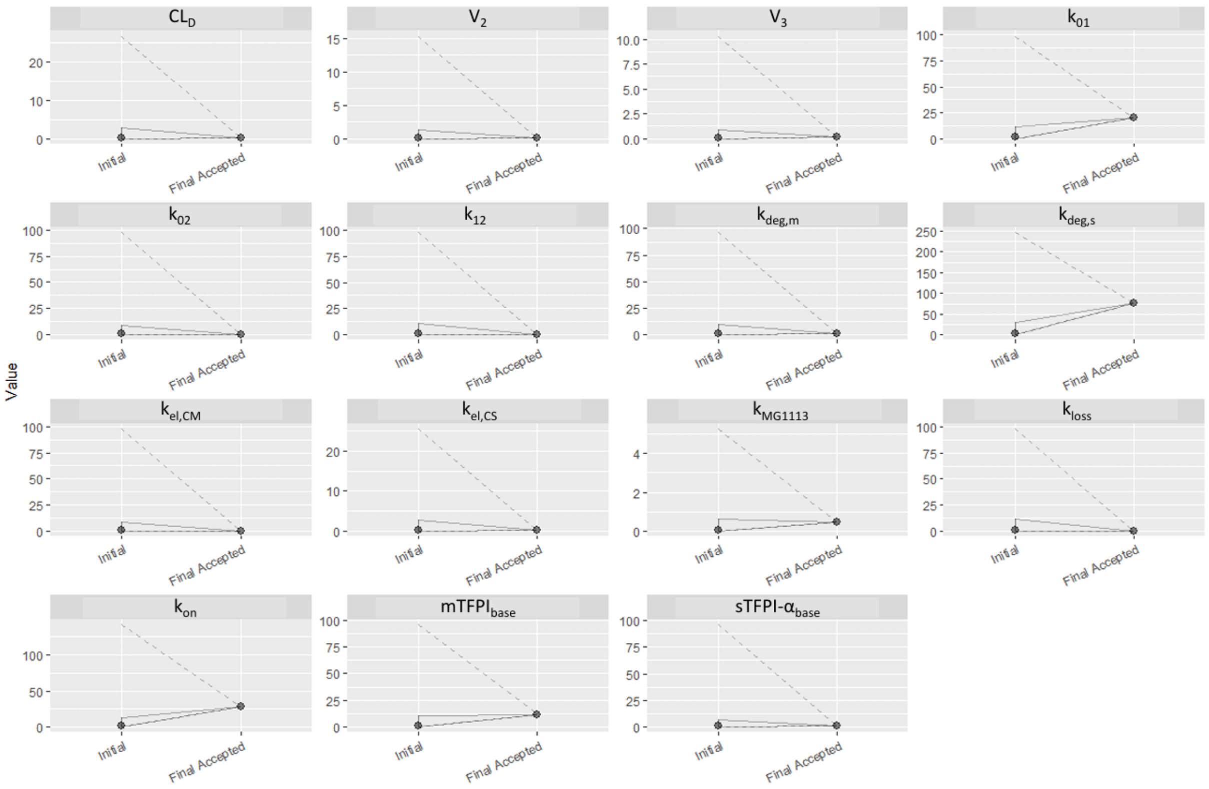

Figure S4. The distribution of the initial and accepted parameter sets from CGNM runs of the refined target-mediated drug disposition model for MG1113. Median values are represented by closed circles, with quartile values indicated by dotted lines. Refer to the text and Table 1 for abbreviations and detailed descriptions.

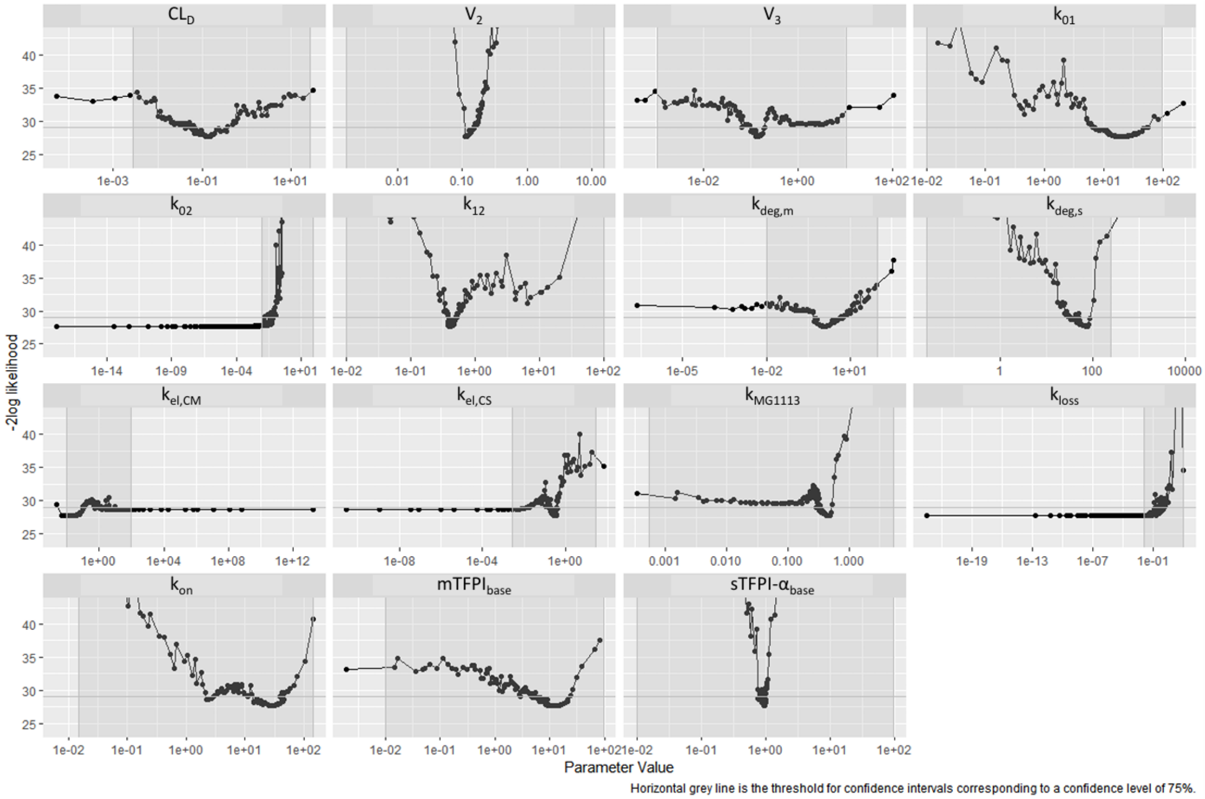

Figure S5. The plot of approximate profile likelihoods of the refined target-mediated drug disposition model for MG1113 and sTFPI-α. The black solid lines and dots represent the approximate profile likelihood calculated via CGNM. Vertical grey lines are the initial ranges of each parameter. Refer to the text and Table 1 for abbreviations and detailed descriptions.

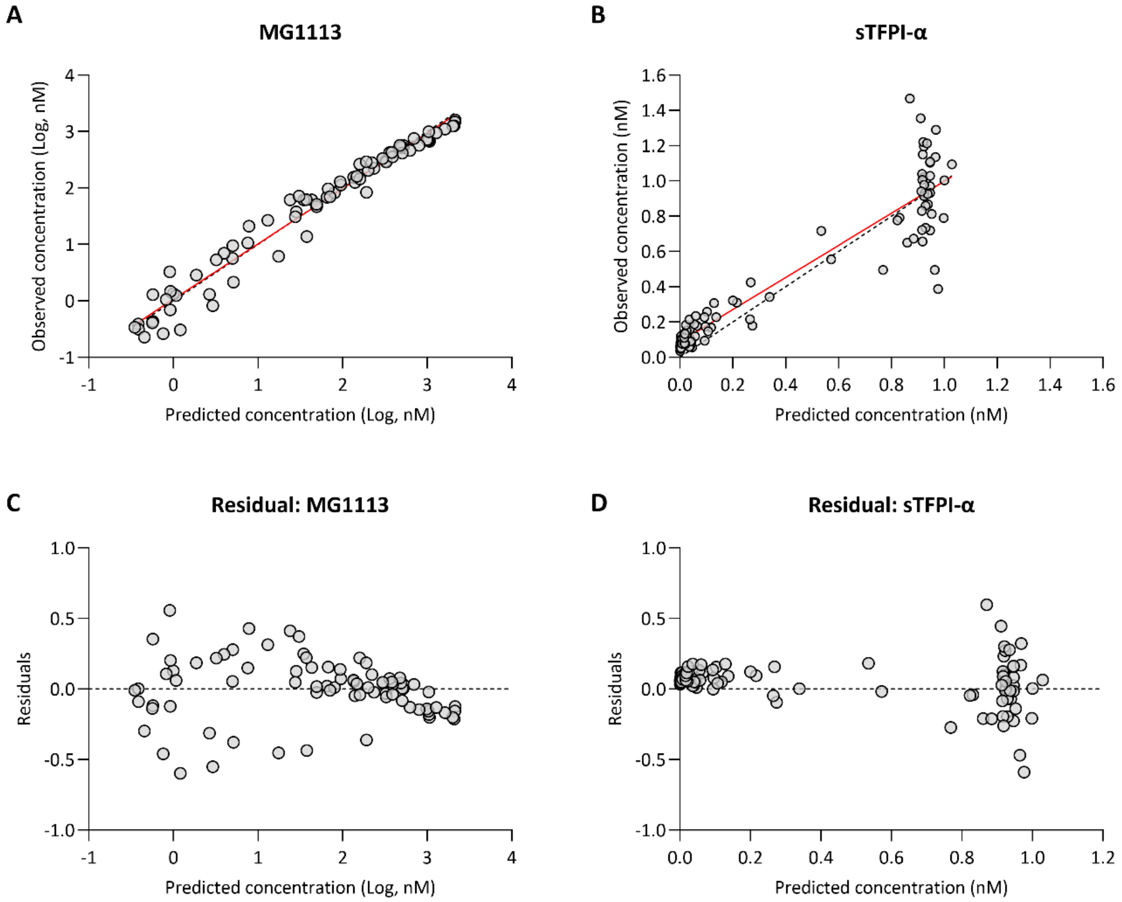

Figure S6. Goodness-of-fit plots for MG1113 and sTFPI-α. The plots compare the mean of observed data and predicted concentrations of MG1113 (A) and sTFPI-α (B). The red line represents the linear regression plot of the mean observed concentrations versus the predicted concentrations. The black dotted line represents the $y = x$ line graph. The plot of residuals versus the predicted concentration of MG1113 (C) and sTFPI-α (D).

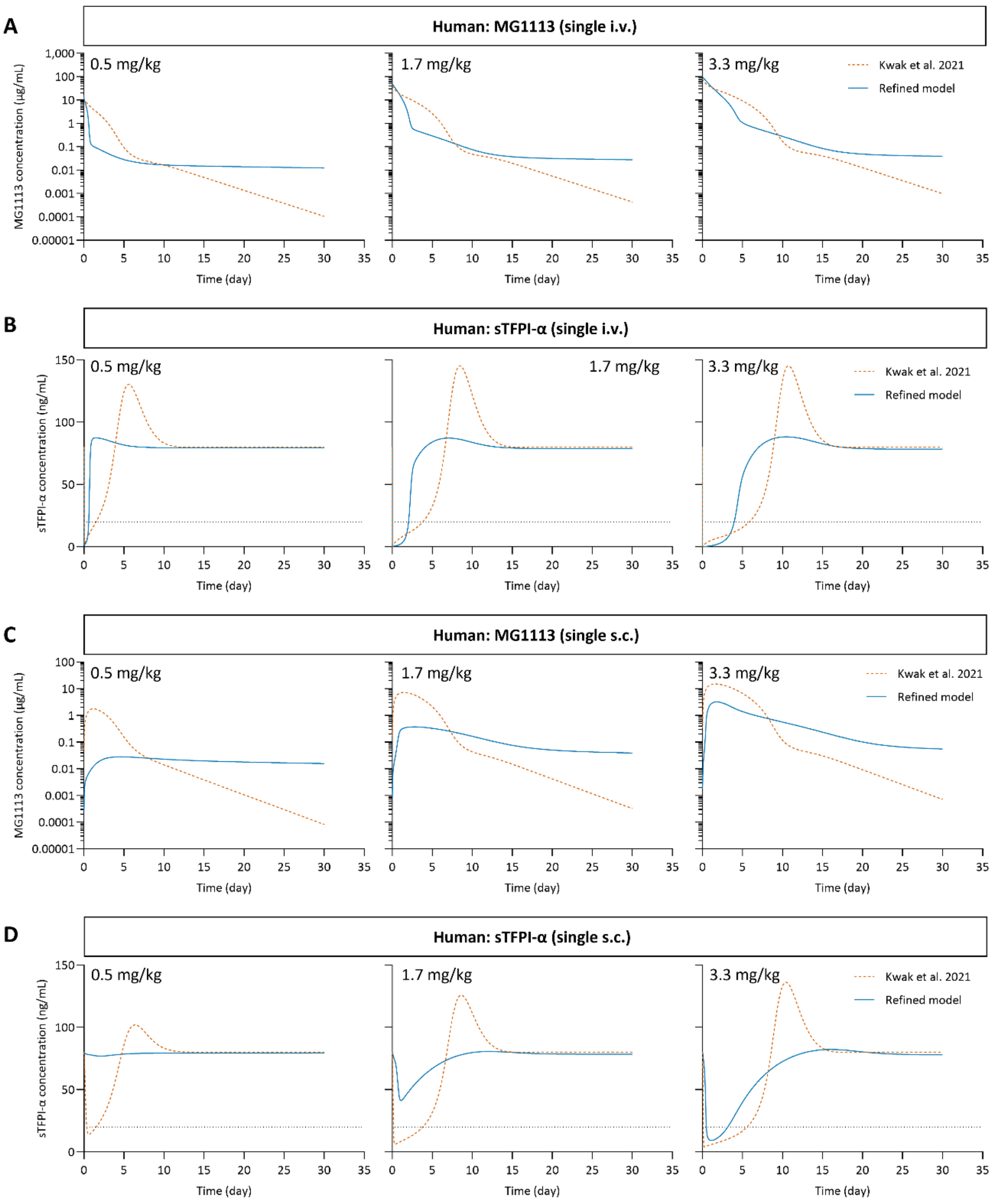

Figure S7. Comparison of results between the previous and refined models for human prediction in a single administration. The plasma concentration-time profiles of MG1113 (A, C) and sTFPI-α (B, D) after a single i.v. or s.c. administration at 0.5, 1.7, and 3.3 mg/kg. (B, D) The horizontal dotted line indicates 25% of the baseline level of sTFPI-α. sTFPI-α, soluble tissue factor pathway inhibitor alpha.

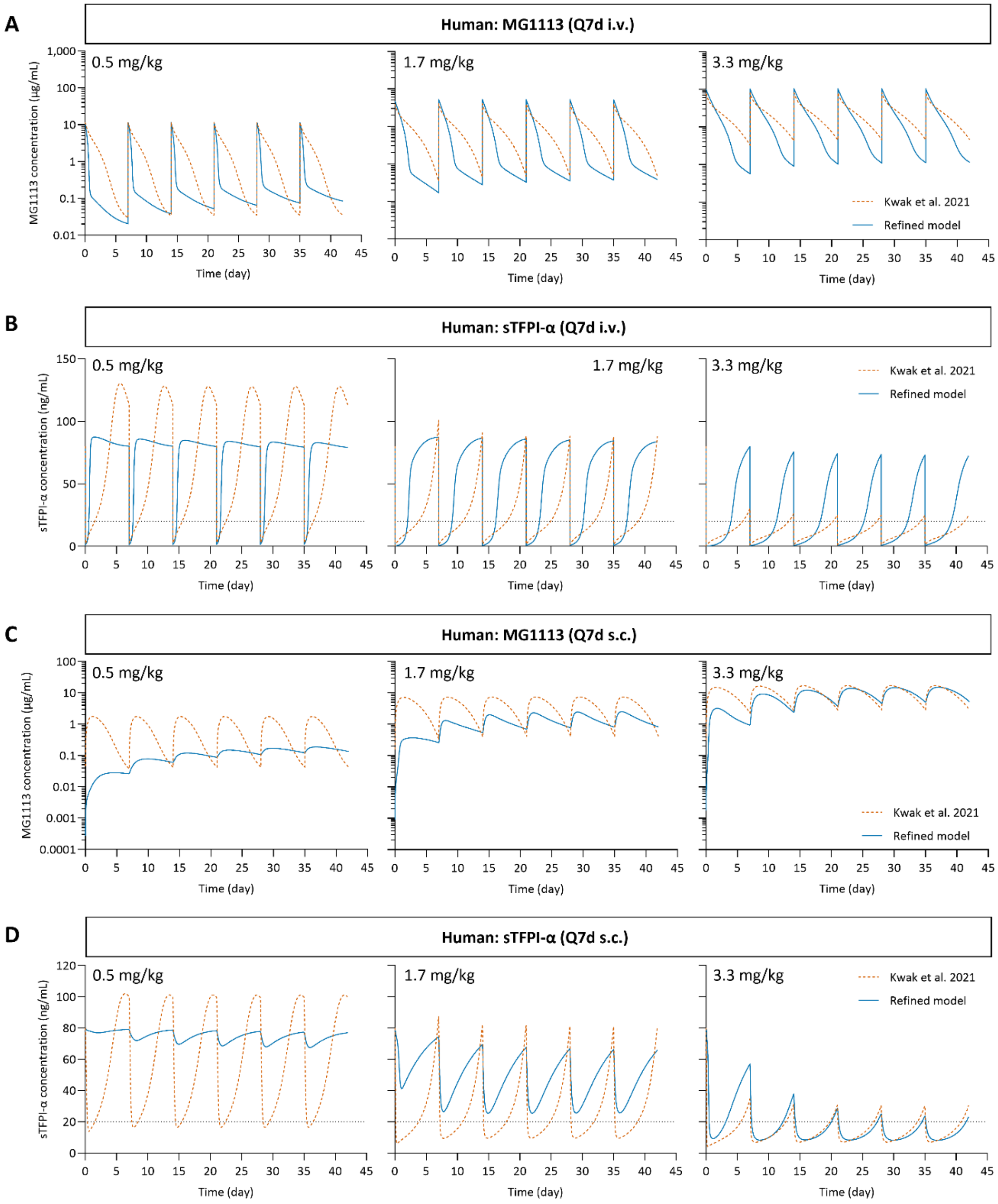

Figure S8. Comparison of results between the previous and refined models for human prediction in multiple administrations. The plasma concentration-time profiles of MG1113 (A, C) and sTFPI-α (B, D) after i.v. or s.c. administration once every 7 days (Q7d) at doses of 0.5, 1.7, and 3.3 mg/kg. (B, D) The horizontal dotted line indicates 25% of the baseline level of sTFPI-α. sTFPI-α, soluble tissue factor pathway inhibitor alpha.

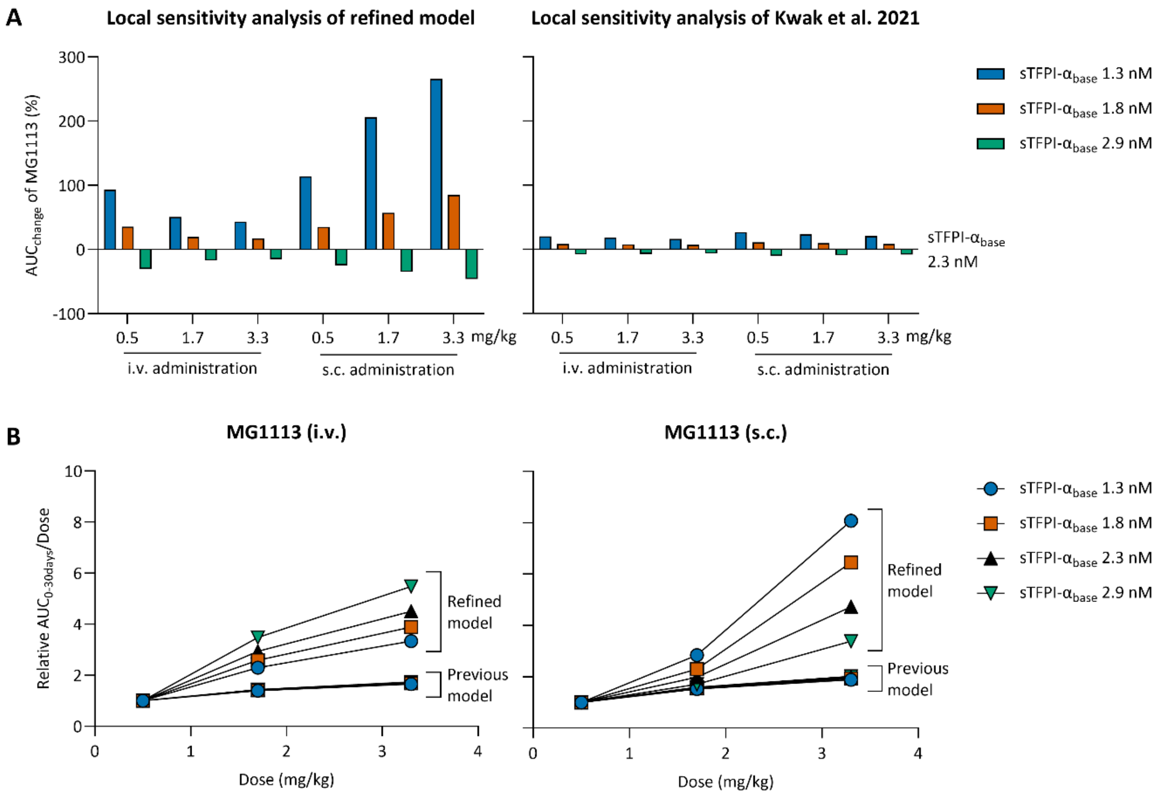

Figure S9. Comparison of the local sensitivity analysis between the previous and refined models for the baseline concentration of soluble tissue factor pathway inhibitor alpha (sTFPI-α_{base}) in human prediction. (A) Relative changes in the predicted $AUC_{0\text{-}30days}$ of MG1113 in humans, in response to changes in the values of sTFPI-α_{base}. (B) Using either the previous model or the refined model, dose-normalized $AUC_{0\text{-}30days}$ were predicted for humans at three doses at various sTFPI-α_{base}. The plots show relative changes in dose-normalized $AUC_{0\text{-}30days}$ compared to the value at 0.5 mg/kg.

$$Relative\ AUC_{0\text{-}30dyas}/Dose = \frac{AUC_{0\text{-}30days,Dose} \div Dose}{AUC_{0\text{-}30days,0.5mg/kg} \div 0.5\ mg/kg}$$

3.2 Supplementary Tables

Table S1. Profile likelihood confidence interval for the model regarding MG1113. The interquartile ranges indicate the upper or lower limits based on the likelihood of the profile.

Parameter (unit)	25%	Best-fit	75%	Identifiability
CL_D (L/day)	0.0403	0.1454	0.3648	Identifiable
V_2 (L)	0.1078	0.1112	0.1528	Identifiable
V_3 (L)	0.0961	0.1346	0.1965	Identifiable
k_{01} (1/day)	6.964	20.32	50.33	Identifiable
k_{02} (1/day)	$< 1.126\times10^{-18}$	NA	0.0757	Non-identifiable
k_{12} (1/day)	0.3704	0.4033	0.5179	Identifiable
$k_{deg,m}$ (1/day)	0.5139	1.150	5.408	Identifiable
$k_{deg,s}$ (1/day)	32.20	75.50	88.61	Identifiable
$k_{el,CM}$ (1/day)	2.854×10^{-3}	NA	$> 1.747\times10^{13}$	Non-identifiable
$k_{el,CS}$ (1/day)	$< 2.942\times10^{-11}$	NA	0.3829	Non-identifiable
$k_{el,MG1113}$ (1/day)	0.3198	0.4543	0.5232	Identifiable
k_{loss} (1/day)	$< 2.886\times10^{-24}$	NA	0.9483	Non-identifiable
k_{on} (1/(nM×day))	2.165	28.50	45.82	Non-identifiable
$mTFPI_{base}$ (nM)	4.799	12.04	22.94	Identifiable
sTFPI-α_{base} (nM)	0.7625	0.9456	1.0023	Identifiable

Refer to the text and Table 1 for other abbreviations.

Table S2. Summary of the CGNM analysis results of the two models.

Optimized Parameters	Refined model<br>• min SSR: 6.34<br>• Accepted parameter set: 136 EA				$k_{02} = k_{loss} = 0$<br>• min SSR: 6.34<br>• Accepted parameter set: 155 EA			
	**Rank 1**	**Min**	**Max**	**Median**	**Rank 1**	**Min**	**Max**	**Median**
K_D (nM)	0.04665				0.04665			
CL_D (L/day)	0.1454	0.1453	0.1458	0.1456	0.1456	0.1454	0.1458	0.1455
V_2 (L)	0.1112	0.1111	0.1113	0.1112	0.1112	0.1112	0.1113	0.1112
V_3 (L)	0.1346	0.1346	0.1348	0.1346	0.1346	0.1346	0.1348	0.1346
k_{01} (1/day)	20.32	20.26	20.37	20.30	0.4035	0.4032	0.4036	0.4034
k_{02} (1/day)	8.859×10^{-8}	3.052×10^{-10}	9.553×10^{-6}	1.549×10^{-7}	0			
k_{12} (1/day)	0.4033	0.4033	0.4037	0.4034	20.30	20.24	20.35	20.30
$k_{deg,m}$ (1/day)	1.150	1.146	1.153	1.151	1.149	1.147	1.155	1.151
$k_{deg,s}$ (1/day)	75.50	75.44	75.56	75.52	75.52	75.44	75.58	75.52
$k_{el,CM}$ (1/day)	0.006851	0.006579	0.006945	0.006848	0.006702	0.006640	0.007123	0.006825
$k_{el,CS}$ (1/day)	0.3094	0.3090	0.3096	0.3093	0.3094	0.3088	0.3096	0.3093
$k_{el,MG1113}$ (1/day)	0.4543	0.4540	0.4550	0.4542	0.4542	0.4540	0.4545	0.4542
k_{loss} (1/day)	3.149×10^{-7}	1.408×10^{-12}	1.141×10^{-5}	4.419×10^{-7}	0			
k_{on} (1/(nM×day))	28.50	28.42	28.52	28.49	28.47	28.44	28.53	28.49
$mTFPI_{base}$ (nM)	12.04	12.03	12.09	12.05	12.05	12.02	12.08	12.05
$sTFPI\text{-}\alpha_{base}$ (nM)	0.9456	0.9454	0.9457	0.9455	0.9456	0.9454	0.9457	0.9455

Refer to the text and Table 1 for other abbreviations.

Table S3. The predicted AUC using the refined model in a scenario of single and multiple administrations of MG1113 to humans.

Route	Dose (mg/kg)	Single administration	
		$AUC_{0\text{-}30days}$ (day×μg/mL)	Dose-normalized $AUC_{0\text{-}30days}$ (day×μg/mL/dose)
i.v.	0.5	4.018	8.036
	1.7	40.16	23.62
	3.3	119.4	36.18
s.c.	0.5	0.5931	1.186
	1.7	4.005	2.36
	3.3	18.52	5.61
**Route**	**Dose (mg/kg)**	**Q7d administration**	
		$AUC_{\tau,ss}$ (day×μg/mL)	Dose-normalized $AUC_{\tau,ss}$ (day×μg/mL/dose)
i.v.	0.5	4.578	9.156
	1.7	45.93	27.02
	3.3	138.2	41.87
s.c.	0.5	1.159	2.318
	1.7	11.07	6.513
	3.3	78.80	23.88

$AUC_{0\text{-}30days}$, the area under the curve from 0 day to 30 days; $AUC_{\tau,ss}$, area under the curve over a dosing interval at steady state; Q7d, every seven days; i.v., intravenous; s.c., subcutaneous

Table S4. Comparison of predicted and observed AUC_{last} values of MG1113 and sTFPI-α from the previous and refined models using cynomolgus monkey data.

Article	Route	Dose (nmol/kg)	AUC_{last} (day × nM)				
			Observed	Predicted		SMAPE (%)	
				Previous	Refined	Previous	Refined
MG1113	i.v.	17.2	510.4	682.6	459.1	28.9	10.6
		34.4	1199.8	1522.4	1243.2	23.7	3.6
		68.8	2918.5	3364.2	3137.1	14.2	7.2
	s.c.	17.2	274.6	346.3	202.6	23.1	30.2
		34.4	875.4	800.4	858.4	9.0	2.0
		68.8	3233.6	1792.2	2624.5	57.4	20.8
sTFPI-α	i.v.	17.2	22.9	20.5	22.2	11.1	3.2
		34.4	21.1	19.0	20.0	10.5	5.3
		68.8	18.5	17.0	17.5	8.2	5.3
	s.c.	17.2	27.6	20.4	21.1	30.0	26.3
		34.4	13.6	19.1	18.1	33.8	28.8
		68.8	19.0	17.3	15.0	9.2	23.4

SMAPE, symmetric mean absolute percentage error; AUC_{last}, the area under the curve to the last measurable point; sTFPI-α, soluble tissue factor pathway inhibitor alpha; i.v., intravenous; s.c., subcutaneous

4 Supplementary Methods

Model equations

AZ, CZ, and V_Z described the amount, concentration, and volume of each compartment. Z refers to 0 = depot, 1 = transit, 2 = central, and 3 = peripheral. The INIT meant the initial condition for the TMDD model of MG1113. For monkeys, doses refer to the amount of MG1113 administered to a 3.5 kg monkey, with the low, medium, and high doses being 60.2 nmol (17.2 nmol/kg), 120.4 nmol (34.4 nmol/kg), and 240.8 nmol (68.8 nmol/kg), respectively.

Related equations

$\boldsymbol{k_{off}} = K_D \times k_{on}$

$\boldsymbol{k_{syn,s}} = k_{deg,s} \times sTFPI\text{-}\alpha_{base}$

$\boldsymbol{k_{syn,m}} = k_{deg,m} \times mTFPI_{base}$

Intravenous administration

The concentration (nM) of free MG1113 in the central compartment:

$INIT(C2_{MG1113}) = Dose/V_2$

$$\frac{\boldsymbol{dC2_{MG1113}}}{\boldsymbol{dt}} = CL_D \times \frac{C3_{MG1113} - C2_{MG1113}}{V_2} + k_{off} \times (C2_{CM} + C2_{CS}) - k_{on} \times C2_{MG1113} \times (mTFPI + sTFPI\text{-}\alpha) - k_{el,MG1113} \times C2_{MG1113}$$

The concentration (nM) of free membrane-bound TFPI (mTFPI) in the central compartment:

$INIT(mTFPI) = mTFPI_{base}$

$$\frac{\boldsymbol{dmTFPI}}{\boldsymbol{dt}} = k_{syn,m} - k_{deg,m} \times mTFPI + k_{off} \times C2_{CM} - k_{on} \times mTFPI \times C2_{MG1113}$$

The concentration (nM) of free soluble TFPI-α (sTFPI-α) in the central compartment:

$INIT(sTFPI\text{-}\alpha) = sTFPI\text{-}\alpha_{base}$

$$\frac{\boldsymbol{dsTFPI\text{-}\alpha}}{\boldsymbol{dt}} = k_{syn,s} - k_{deg,s} \times sTFPI\text{-}\alpha + k_{off} \times C2_{CS} - k_{on} \times sTFPI\text{-}\alpha \times C2_{MG1113}$$

The concentration (nM) of mTFPI/MG1113 complex in the central compartment:

$INIT(C2_{CM}) = 0$

$$\frac{\boldsymbol{dC2_{CM}}}{\boldsymbol{dt}} = k_{on} \times mTFPI \times C2_{MG1113} - (k_{off} + k_{el,CM}) \times C2_{CM}$$

The concentration (nM) of sTFPI-α/MG1113 complex in the central compartment:

$INIT(C2_{CS}) = 0$

$$\frac{\boldsymbol{dC2_{CS}}}{\boldsymbol{dt}} = k_{on} \times sTFPI\text{-}\alpha \times C2_{MG1113} - (k_{off} + k_{el,CS}) \times C2_{CS}$$

The concentration (nM) of free MG1113 in the peripheral compartment:

$INIT(C3_{MG1113}) = 0$

$$\frac{\boldsymbol{dC3_{MG1113}}}{\boldsymbol{dt}} = CL_D \times \frac{C2_{MG1113} - C3_{MG1113}}{V_3}$$

<u>Subcutaneous administration</u>

The amount (nmol) of free **MG1113** in the depot compartment:

$INIT(A0_{MG1113}) = Dose\ (nmol)$

$$\frac{\boldsymbol{dA0_{MG1113}}}{\boldsymbol{dt}} = -(k_{01} + k_{02} + k_{loss}) \times A0_{MG1113}$$

The amount (nmol) of free MG1113 in the transit compartment:

$INIT(A1_{MG1113}) = 0$

$$\frac{\boldsymbol{dA1_{MG1113}}}{\boldsymbol{dt}} = k_{01} \times A0_{MG1113} - k_{12} \times A1_{MG1113}$$

The concentration (nM) of free MG1113 in the central compartment:

$INIT(C2_{MG1113}) = 0$

$$\frac{\boldsymbol{dC2_{MG1113}}}{\boldsymbol{dt}} = \frac{k_{02} \times A0_{MG1113} + k_{12} \times A1_{MG1113} + CL_D \times (C3_{MG1113} - C2_{MG1113})}{V_2} + k_{off} \times (C2_{CM} + C2_{CS}) - k_{on} \times C2_{MG1113} \times (mTFPI + sTFPI\text{-}\alpha) - k_{el,MG1113} \times C2_{MG1113}$$

The ODEs for the concentration of mTFPI, sTFPI-α, the sTFPI-α/MG1113 complex ($C2_{CS}$), and mTFPI/MG1113 complex ($C2_{CM}$) in the central compartment and the free MG1113 in the peripheral compartment ($C3_{MG1113}$) were the same as those for intravenous administration.

5 References

1. Kwak EY, Kim MJ, Park JH, Jung HW, Jung ME. Target-mediated drug disposition modeling of an anti-TFPI antibody (MG1113) in cynomolgus monkeys to predict human pharmacokinetics and pharmacodynamics. J Thromb Haemost. 2021;19(6):1425-35.

2. Girard TJ, Tuley E, Broze GJ, Jr. TFPIbeta is the GPI-anchored TFPI isoform on human endothelial cells and placental microsomes. Blood. 2012;119(5):1256-62.

3. Straube R. Target-Mediated Drug Disposition (TMDD) Revisited: High Versus Low-Affinity Approximations of the TMDD Model. CPT Pharmacometrics Syst Pharmacol. 2025;14(7):1262-72.

4. Yan X, Mager DE, Krzyzanski W. Selection between Michaelis-Menten and target-mediated drug disposition pharmacokinetic models. J Pharmacokinet Pharmacodyn. 2010;37(1):25-47.

5. Dua P, Hawkins E, van der Graaf PH. A Tutorial on Target-Mediated Drug Disposition (TMDD) Models. CPT Pharmacometrics Syst Pharmacol. 2015;4(6):324-37.

6. Shah DK, Betts AM. Towards a platform PBPK model to characterize the plasma and tissue disposition of monoclonal antibodies in preclinical species and human. J Pharmacokinet Pharmacodyn. 2012;39(1):67-86.

7. Neuber T, Frese K, Jaehrling J, Jager S, Daubert D, Felderer K, et al. Characterization and screening of IgG binding to the neonatal Fc receptor. MAbs. 2014;6(4):928-42.

8. Maroney SA, Ellery PE, Mast AE. Alternatively spliced isoforms of tissue factor pathway inhibitor. Thromb Res. 2010;125 Suppl 1:S52-6.

9. Yuan D, Rode F, Cao Y. A systems pharmacokinetic/pharmacodynamic model for concizumab to explore the potential of anti-TFPI recycling antibodies. Eur J Pharm Sci. 2019;138:105032.

10. Sanchez-Felix M, Burke M, Chen HH, Patterson C, Mittal S. Predicting bioavailability of monoclonal antibodies after subcutaneous administration: Open innovation challenge. Adv Drug Deliv Rev. 2020;167:66-77.

11. Viola M, Sequeira J, Seica R, Veiga F, Serra J, Santos AC, et al. Subcutaneous delivery of monoclonal antibodies: How do we get there? J Control Release. 2018;286:301-14.
